# The PHP domain of PolX from *Staphylococcus aureus* aids high fidelity DNA synthesis through the removal of misincorporated deoxyribo-, ribo- and oxidized nucleotides

**DOI:** 10.1038/s41598-021-83498-1

**Published:** 2021-02-18

**Authors:** Shilpi Nagpal, Deepak T. Nair

**Affiliations:** 1 National Centre for Biological Sciences, Tata Institute of Fundamental Research, UAS-GKVK Campus, Bellary Road, Bangalore, 560065 India; 2 Regional Centre for Biotechnology, NCR Biotech Science Cluster, 3rd Milestone, Faridabad-Gurgaon Expressway, P.O. Box No. 3, Faridabad, Haryana 121001 India

**Keywords:** Biochemistry, Molecular biology

## Abstract

The X family is one of the eight families of DNA polymerases (dPols) and members of this family are known to participate in the later stages of Base Excision Repair. Many prokaryotic members of this family possess a Polymerase and Histidinol Phosphatase (PHP) domain at their C-termini. The PHP domain has been shown to possess 3′–5′ exonuclease activity and may represent the proofreading function in these dPols. PolX from *Staphylococcus aureus* also possesses the PHP domain at the C-terminus, and we show that this domain has an intrinsic Mn^2+^ dependent 3′–5′ exonuclease capable of removing misincorporated dNMPs from the primer. The misincorporation of oxidized nucleotides such as 8oxodGTP and rNTPs are known to be pro-mutagenic and can lead to genomic instability. Here, we show that the PHP domain aids DNA replication by the removal of misincorporated oxidized nucleotides and rNMPs. Overall, our study shows that the proofreading activity of the PHP domain plays a critical role in maintaining genomic integrity and stability. The exonuclease activity of this enzyme can, therefore, be the target of therapeutic intervention to combat infection by methicillin-resistant-*Staphylococcus-aureus*.

## Introduction

DNA Polymerases (dPols) have been classified into eight families, the A, B, C, D, X, Y, reverse transcriptase and PrimPol on the basis of sequence/structural similarity of the domains which catalyze the nucleotide polymerization reaction^1–4^. Among these families, the X- family DNA polymerases (PolX) are present in many organisms with representatives reported in archaea, viruses, bacteria, and eukaryotes^4–11^. These enzymes are generally involved in the DNA synthesis step during base excision repair (BER)^12–16^ and non-homologous end joining (NHEJ) pathway in double-strand break (DSB) repair^17–19^ by virtue of their gap-filling mechanism^20,21^. The mammalian PolX members share similar architecture which is composed of mainly two domains: a BRCA1 C-terminal (BRCT) as N-terminus domain and a PolX core (PolXc) at C- terminus^22–24^ except for Polβ which consist only of PolXc. The mammalian PolXc domain possesses the polymerase activity and it contains two signature helix-hairpin-helix motifs (HhH) that helps in the interaction of the polymerase with the DNA^22^. All the eukaryotic X family polymerases which have been reported so far lack 3′–5′ exonuclease (proofreading) activity^25^.

Sequence analysis of the bacterial PolXs unveiled that none of them retained the BRCT domain, and many of them have acquired a Polymerase and Histidinol Phosphatase (PHP) domain at the C-terminus^6,7^. Also, in bacterial and archaeal PolXs, a third conserved HhH motif is found in the fingers subdomain and has a role in the correct positioning of primer-terminus of the gapped DNA at the polymerization and PHP active site^26^. The PHP domain generally adopts a distorted (βα)_7_ barrel topology^27–29^. In the case of PolX from *T. thermophilus* (ttPolX), it has been shown that the enzyme possesses Mn^2+^ dependent AP endonuclease^14^ and 3′–5′ exonuclease activity in addition to Mg^2+^/Mn^2+^ dependent polymerase activity^6^. The X family polymerase in bacterium *B. subtilis* (bsPolX) also possess Mg^2+^/Mn^2+^ dependent polymerization activity and Mn^2+^ dependent exonuclease and AP endonuclease activities^26,30^. The biochemical characterization of PolX from *D. radiodurans* (drPolX) shows that this enzyme also has Mg^2+^/Mn^2+^ dependent polymerase activity and Mn^2+^ metal ion-dependent 3′–5′ exonuclease activity like other two characterized bacterial PolXs^31^. For ttPolX and bsPolX, the catalytic residues critical for exonuclease activity are present at the C-terminal PHP domain^6,32,33^. The studies on bsPolX show that the residues present in the third HhH motif in the fingers subdomain stabilize the DNA substrate in the PHP domain and affect its exonuclease activity^26,30,32^. In contrast in the case of drPolX, the exonuclease activity is located in the PolXc domain and residues in the PHP domain contribute towards DNA structure based specificity^31^.

Despite the high fidelity exhibited by dPols, ribonucleotide triphosphates (rNTPs) are incorporated into the genome due to the relatively high cellular concentration of rNTPs as compared to deoxyribonucleotide triphosphates (dNTPs)^34^. Additionally, it has been suggested that during the repair of DSBs by employing the NHEJ pathway, rNTPs are incorporated by repair dPols, both in mammals^35^ as well as in bacterial systems^36^. Members of X-family dPols exhibit the insertion of rNTPs during NHEJ^35^. The mammalian representatives such as Polμ^35^ and TdT^37^ incorporate rNTPs with low discrimination, whereas Polβ^38^ and Polλ^39,40^ incorporate rNTPs less efficiently. In the case of bacterial X-family polymerases, ttPolX shows lower rNTPs incorporation as compared to dNTPs^6^.

Reactive oxygen species (ROS)^41^ arise in prokaryotes through intrinsic metabolic pathways^42^ or due to the impaired respiration^43–45^ caused by external agents such as antibiotics^46–48^. ROS are known to oxidize the nucleotide pool^49–51^ and give rise to damaged nucleotides such as 8-oxo-2′-deoxyguanosine triphosphate (8oxodGTP) and 2-hydroxy-2′-deoxyadenosine triphosphate (2-OH-dATP)^52^. The 8oxodGMP nucleotide forms Watson–Crick base pair with dC and a Hoogsteen base pair with dA in the dPol active site. 2-OH-dATP can be misincorporated by DNA polymerases opposite guanine in template DNA during DNA replication. Due to this dual coding potential of 8oxodGMP and 2-OH-dAMP, misinsertions by dPol may lead to A:T → C:G and G:C → T:A transversions, respectively^53–55^. The repair polymerases from X family like human Polβ^56–58^, Polµ^59,60^ and Polλ^61^ which are involved in DNA repair pathways like BER and NHEJ, exhibit significant ability to incorporate 8oxodGTP^62^. However, in the case of bacterial X family polymerase, both ttPolX and bsPolX show low frequency of incorporation of 8oxodGTP irrespective of the templating nucleotide. ttPolX and bsPolX incorporate 8oxodGTP opposite dA with 15- and 218-times lower efficiency, respectively than dTTP opposite dA^63,64^.

To shed further light on the DNA polymerase and exonuclease activities of bacterial X-family dPols, we have carried out the biochemical characterization of PolX from *S. aureus* (saPolX). Like other bacterial PolXs, it has DNA- dependent DNA polymerase activity which is present in its N-terminal and 3′–5′ exonuclease activity located in the PHP domain at the C terminus. We have cloned, expressed and purified the saPolX to high purity and homogeneity. In addition, based on sequence homology, we have prepared a mutant version of the enzyme that lacks in exonuclease activity. A comparison of the DNA polymerase activities of the wt-saPolX and mutant versions showed that the exo- construct exhibited substantial ability to incorporate mismatches, ribonucleotides, and oxidized nucleotides. We observed that both Mg^2+^ and Mn^2+^ can act as metal ion cofactors for the exonuclease activity. Further experiments showed that the exonuclease activity is capable of removing mismatches, ribonucleotides, and oxidized nucleotides present at the primer terminus. Overall, our studies show that the PHP domain of PolX from *S. aureus* aids high fidelity DNA synthesis through the removal of misincorporated deoxyribo-, ribo-, and oxidized nucleotides.

## Results

### Homology modelling and structural features of saPolX

**Figure 1:**
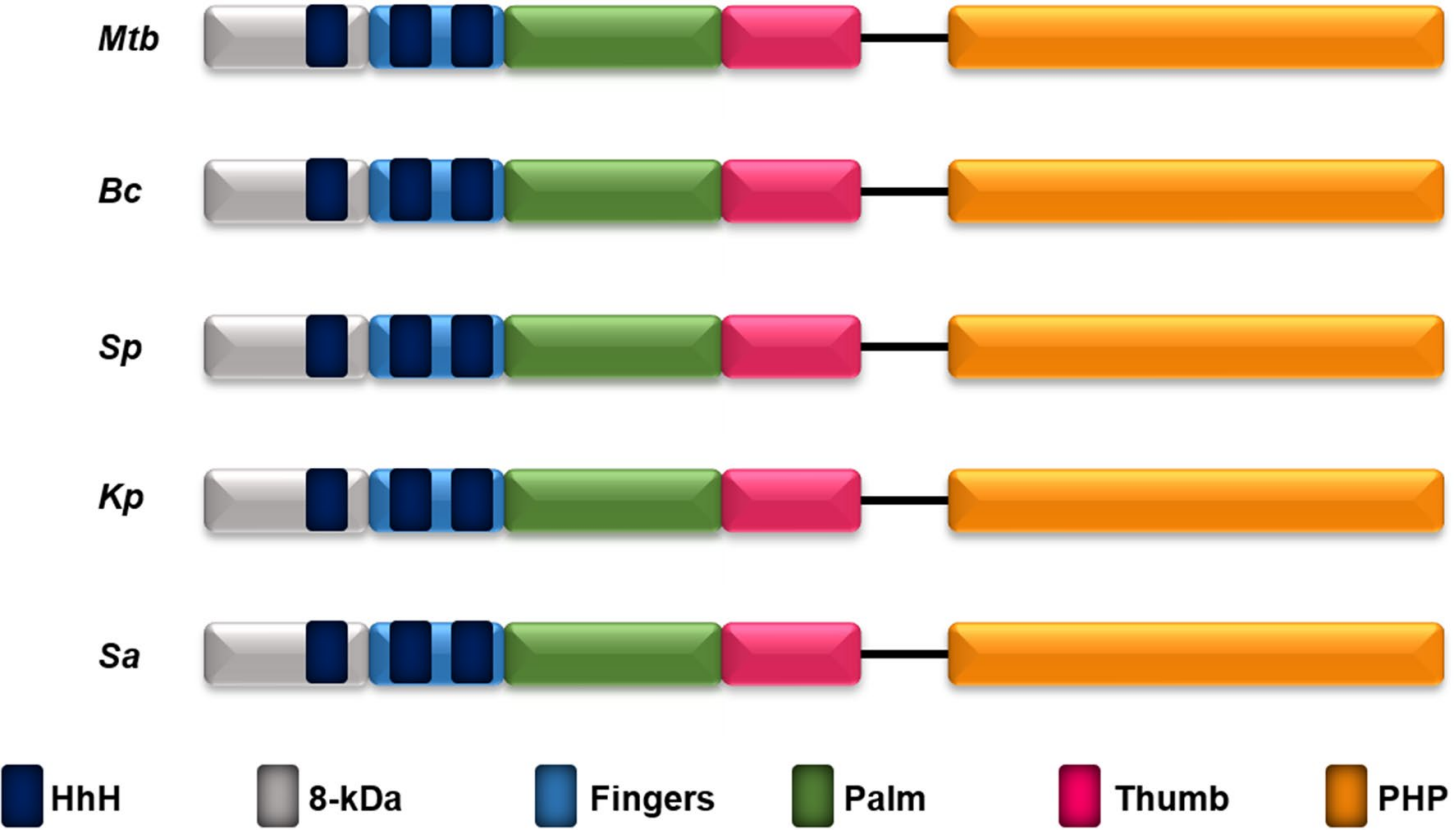
Domain organization of selected members of PolX from pathogenic bacteria. PolX in all the compared bacteria has conserved 8-kDa domain, HhH motifs, fingers, palm, thumb subdomains and PHP domain which are displayed in grey, dark blue, light blue, green, pink and orange color, respectively. The NCBI GenBank accession numbers and abbreviations of the organisms compared are: *Mycobacterium tuberculosis* (CND95738.1) *(Mtb), Bacillus cereus* (AAP11460.1) *(Bc), Streptococcus pneumoniae* (COE05251.1) *(Sp), Klebsiella pneumonia* (PCQ20452.1) *(Kp) and Staphylococcus aureus* (AAW38032.1) *(Sa)*.

**Figure 2:**
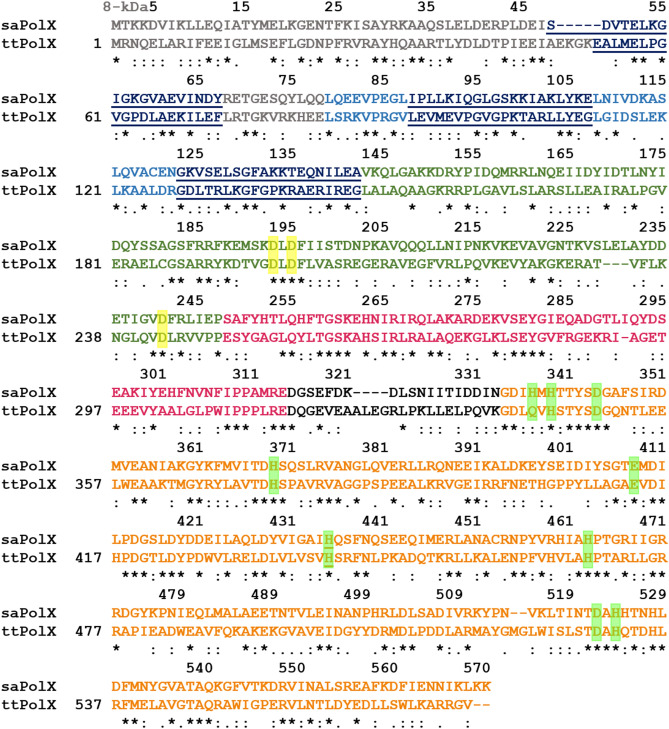

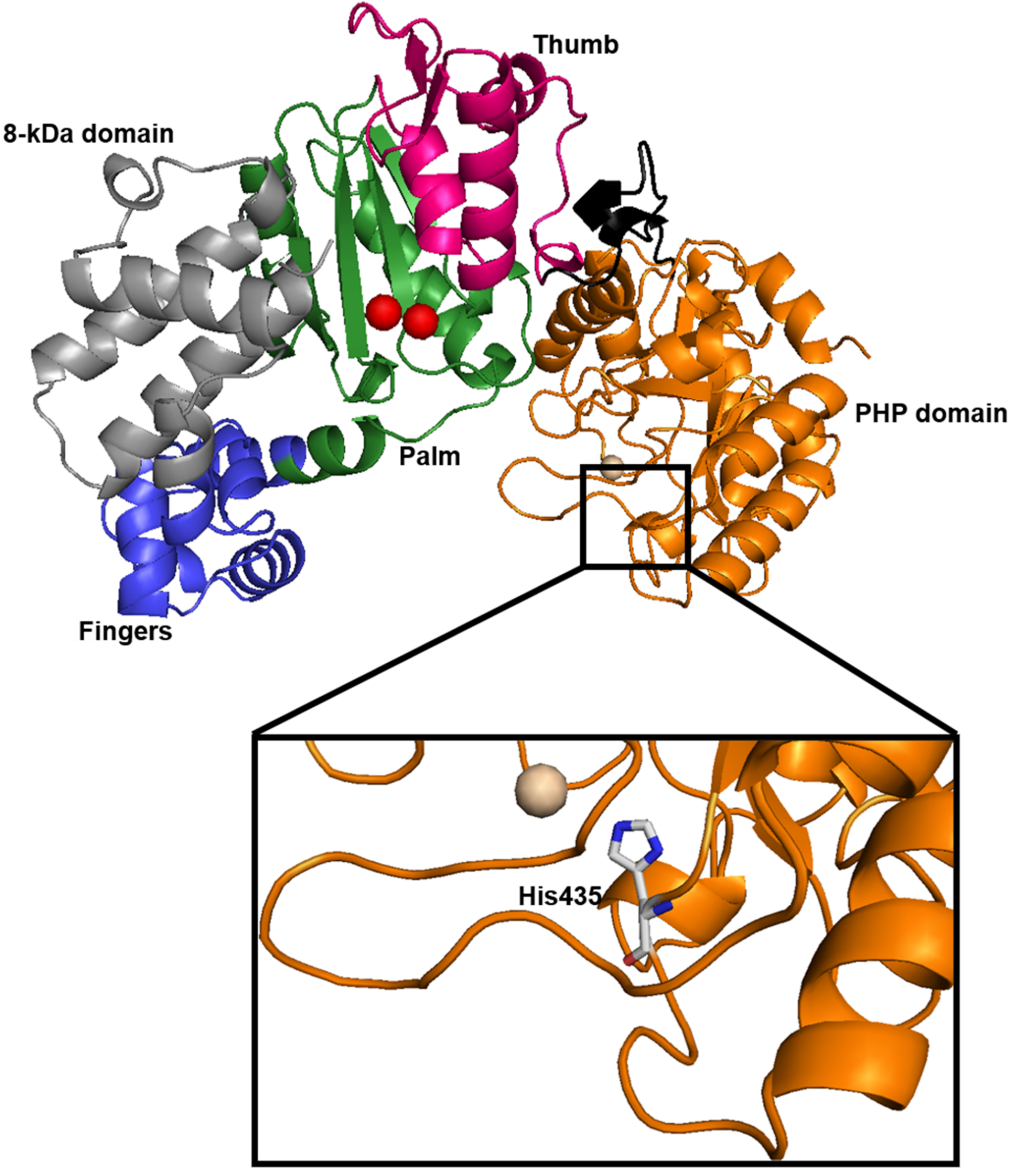
Sequence alignment of saPolX with ttPolX and homology model of saPolX. (**A**) The proteins encoded by genes *SACOL1153* (saPolX) and *TTHA1150* (ttPolX) from organisms *S. aureus* and *T. thermophilus,* respectively are aligned and compared. The residues in grey color represent the conserved 8-kDa domain, light blue color residues show fingers, green color shows palm, pink-colored residues show thumb and orange residues show PHP domain. The residues in three conserved HhH motifs are shown in dark blue color and underlined. The catalytic residues critical for DNA polymerase activity (including D193) of saPolX are highlighted by yellow boxes. The nine residues highlighted in green boxes indicate the residues, including H435 (underlined) that are critical for the exonuclease activity of ttPolX. (**B**) Homology model for saPolX. The homology model for saPolX was created using ttPolX binary structure as a template (PDB code: 3AU2) at the SWISS-MODEL server. The conserved 8-kDa, fingers, palm, thumb and PHP domains are displayed in grey, light blue, green, pink and orange color. The inset figure shows a close up of the PHP domain with the H435 displayed in stick representation and colored according to the atom. The H435 residue is known to be important for exonuclease activity and was selected for substitution with Ala (H435A) to prepare a mutant version that is incapable of exonuclease activity.

PSI-BLAST^65^ searches suggested the presence of PolX in low-GC content gram-positive pathogenic bacteria *S. aureus*^7^. The sequence analysis of the coding sequence of *S. aureus* subsp*. aureus* COL, complete genome (GenBank: CP000046.1) showed that it encodes for 570 amino acid residues long PolX protein with the calculated molecular mass of 64.87 kDa. Multiple sequence alignment of the protein encoded by gene *SACOL1153* with other conserved members of the PolX family confirmed the presence of structurally conserved PolX like core and a PHP domain (Fig. 1). saPolX shares about 32% sequence identity with ttPolX with 98% sequence coverage. Thus, a homology model of the structure of saPolX was built using the structure of ttPolX^66^ (PDB code: 3AU2) as a template at the SWISS-MODEL server^67^ (Fig. 2A, B). On further analysis, we found that the PolXc domain had all the four subdomains: the N-terminal 8-kDa domain followed by the fingers, palm and thumb which are the signature of right-handed DNA polymerase (Fig. 1). The three HhH motifs which are known for binding with DNA in a sequence-independent manner are conserved in saPolX^68^. Each HhH motif is 20 amino acid residues long stretch with consensus sequence Gh(G/A) where h is a hydrophobic residue. The first conserved HhH motif (GhG/Axxxx) present in the 8-kDa domain of saPolX has GIGKGVA amino acid sequence^29,69^ (Fig. 2A). The second and third HhH motifs present in the fingers subdomain have sequences: GLGSKKI and GFAKKTE, respectively (Fig. 2A)^26,70^. The first and second HhH motifs are shown to interact with the sugar-phosphate backbone of the downstream strand and primer of the 1 nucleotide gapped DNA substrate in ttPolX^66^ and Polβ^71^. The third HhH motif located in the C-terminus of fingers is conserved only in prokaryotic and archeal PolXs. Baños et al. (2012) had shown that it is also involved in the DNA binding, especially it allows the correct localization of the primer-terminus at the PolXc and PHP active sites for polymerization, AP endonuclease activity and 3′–5′ exonuclease activity^26^. The palm subdomain contains three highly conserved catalytic aspartate residues that coordinate with the two divalent cations during the polymerization reaction. In the catalytic mutant D193A of saPolX, we observed the complete loss of polymerization activity (Fig. 4). The thumb subdomain shows significant homology with other prokaryotic and eukaryotic members of the PolX family. The whole PolXc domain is followed by a PHP domain which has been shown to possess 3′–5′ exonuclease activity^6,7^. The structures of ttPolX and drPolX show that the PHP domain forms a distorted (βα)_7_ TIM barrel fold, similar to *E. coli* YcdX which requires three bound metal ions in the active site for catalysis^27,28^. The sequence of saPolX was aligned with ttPolX to identify residues of the PHP domain that are potentially critical for exonuclease activity^6,33^. Based on this analysis, the residue H435 in saPolX was selected for substitution with Ala to create an exo- mutant named H435A henceforth.

### Purification and identification of recombinant saPolX

**Figure 3:**
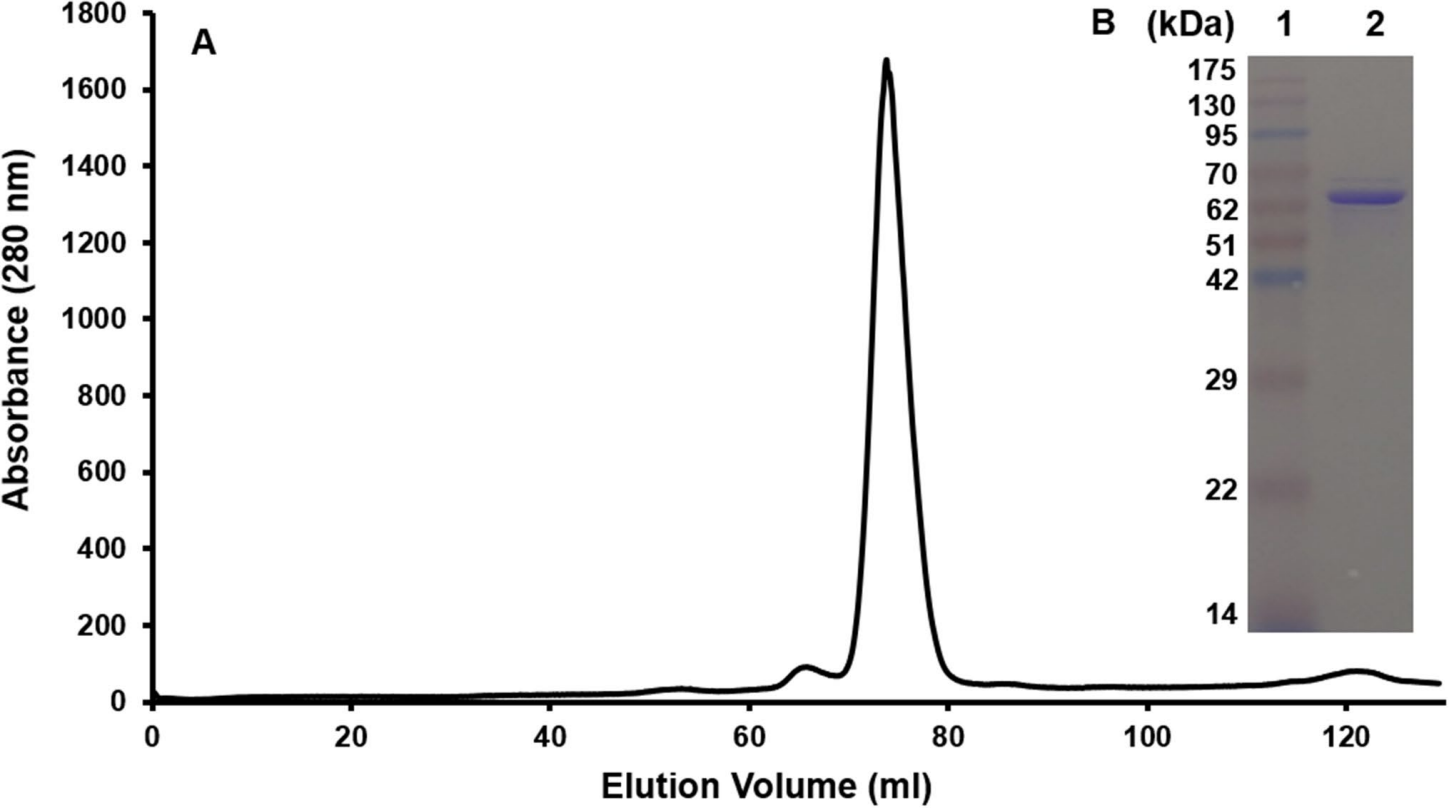
Purification of wt-saPolX and H435A protein. (**A**) The gel filtration chromatogram for wt-saPolX is shown. The gel filtration profile showed the presence of a single peak with V_R_ ~ 74 ml. (**B**) SDS-PAGE analysis of purified wt-saPolX protein showed the presence of a single band corresponding to 66.97 kDa. The H435A and H435A/D193A mutants used in this study were purified with the same protocol as wt-saPolX. The gel filtration chromatogram for mutant proteins also showed the presence of a single peak with V_R_ ~ 74 ml and SDS-PAGE analysis of purified mutants showed the presence of a single band in gel.

The recombinant protein saPolX was overexpressed in *E. coli* BL21-CodonPlus cells. Initially, the protein was purified using His-tag affinity chromatography. The protein was further purified by subjecting it to anion exchange chromatography followed by size-exclusion chromatography. The saPolX protein was eluted in the retention volume (V_R_) ~ 74 ml in 16/600 Superdex 200 column (Fig. 3A). We obtained approximately 7 mg of protein from 5 L of culture. The purity of the sample was estimated by SDS–PAGE where the gels were stained either by Coomassie Brilliant Blue R-250 (Fig. 3B) or by Silver stain (Supplementary Fig. S1). Silver stain can detect 0.25–0.50 ng of protein. For Silver staining, 400 ng of wt-saPolX and H435A mutant proteins were loaded on the SDS-PAGE gel. Both the proteins were found to be more than 99% pure (Supplementary Fig. S1). To confirm the identity of the saPolX, we performed Peptide Mass Fingerprinting (PMF) using Mass spectrometry. For this, we performed the proteolysis of the purified saPolX using trypsin. Under the mild basic conditions, endoprotease trypsin hydrolyzes a protein at the basic amino acid residue sites. The digested sample containing many short peptides was subjected to mass spectrometry and the identified peptides were matched against the specific organism in the NCBI database. The PMF analysis confirmed the purified protein was a PolX from *S. aureus*. To re-confirm the absence of contaminants, the electrospray-ionization mass spectrometry (ESI–MS) was commercially done for the purified wt-saPolX and H435A mutant proteins. The ESI–MS results re-confirmed the absence of any polymerase/nuclease contaminants. The only prominent hit obtained in ESI–MS was PolX from organism *S. aureus*. Once the identity of the protein was confirmed, the plasmids for the single mutant H435A and double mutant H435A/D193A harbouring substitutions to Ala at catalytic sites for polymerase activity (Asp193) and exonuclease activity (His435) were generated using Site-directed mutagenesis. The presence of mutations was confirmed by DNA sequencing of the plasmids and mutant proteins were purified with the same protocol as for the wt-saPolX.

### DNA polymerase and exonuclease activities of saPolX

**Figure 4:**
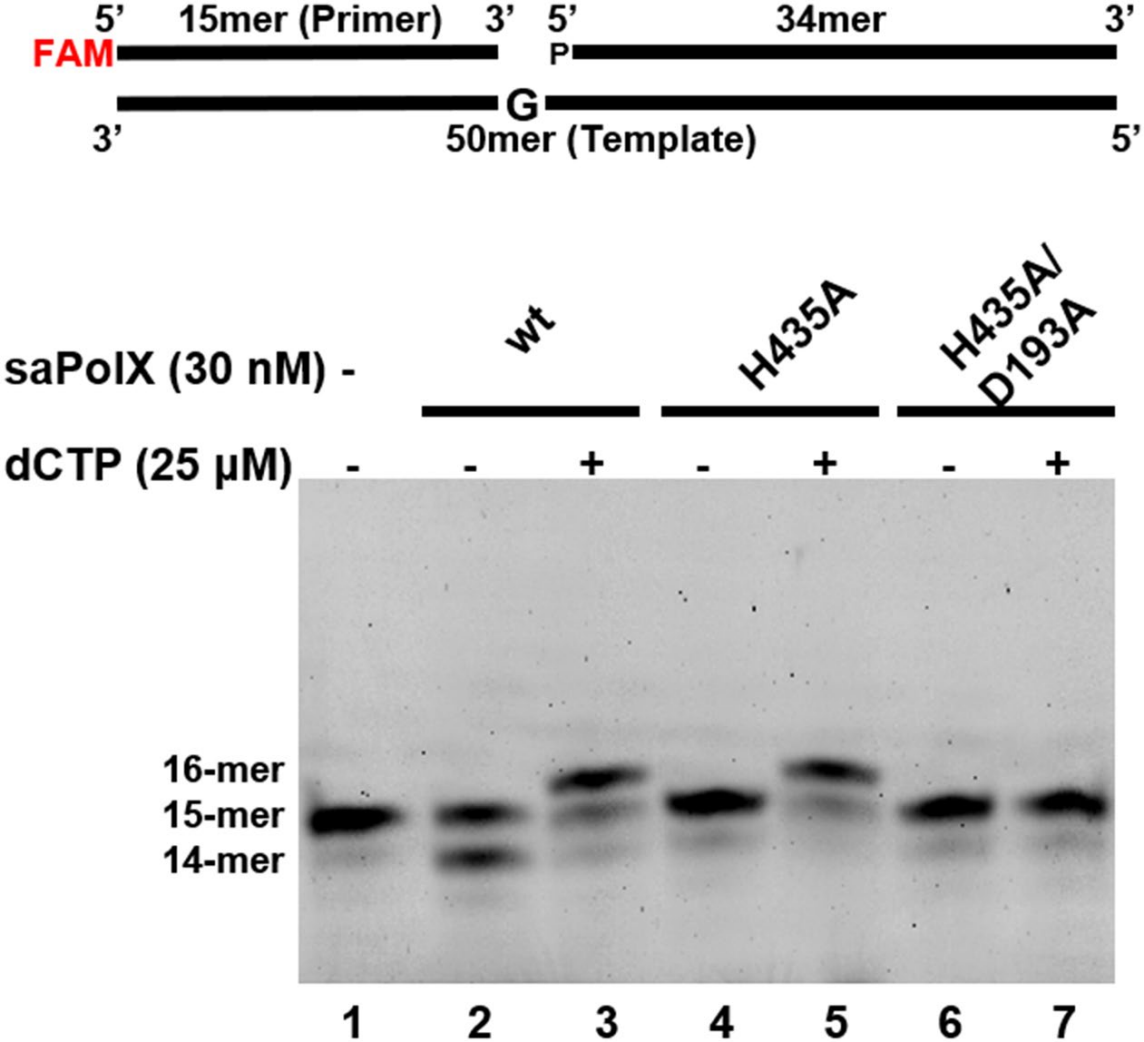
Enzyme activities of wt-saPolX, mutant H435A and double mutant H435A + D193A. Primer extension assays were performed on a gapped DNA substrate to check the polymerase and exonuclease activities present in wt-saPolX and exonuclease mutant H435A. The results show that wt-saPolX possesses both polymerase and exonuclease activities. The mutation H435A abolished the exonuclease activity of saPolX and the mutation D193A removed the polymerase activity.

Primer extension assay showed that wt-saPolX had Mg^2+^ dependent 5′–3′ template-dependent polymerase activity (Fig. 4, lane 3). In the polymerase assay, we observed that the gapped substrate was extended in 5′–3′ direction in the presence of Mg^2+^ (Fig. 4, lane 3). However, we observed that wt-saPolX also showed 3′–5′ exonuclease activity (Fig. 4, lane 2). To corroborate that the activities observed were indeed by wt-saPolX, we performed the polymerase assay and exonuclease activity assay using the catalytic mutants. The H435A exo- mutant was prepared to check that the exonuclease activity was executed by wt-saPolX. The H435A mutant exhibited no exonuclease activity on the gapped DNA substrate. In the case of the double mutant H435A/D193A, the polymerase activity of saPolX was abolished completely, which further confirmed the absence of any co-purified contaminants (Fig. 4). As wt-saPolX executed 3′–5′ exonuclease activity, we compared the biochemical activities of wt-saPolX and H435A to understand the role of PHP domain which resides at the C-termini of the saPolX.

**Figure 5:**
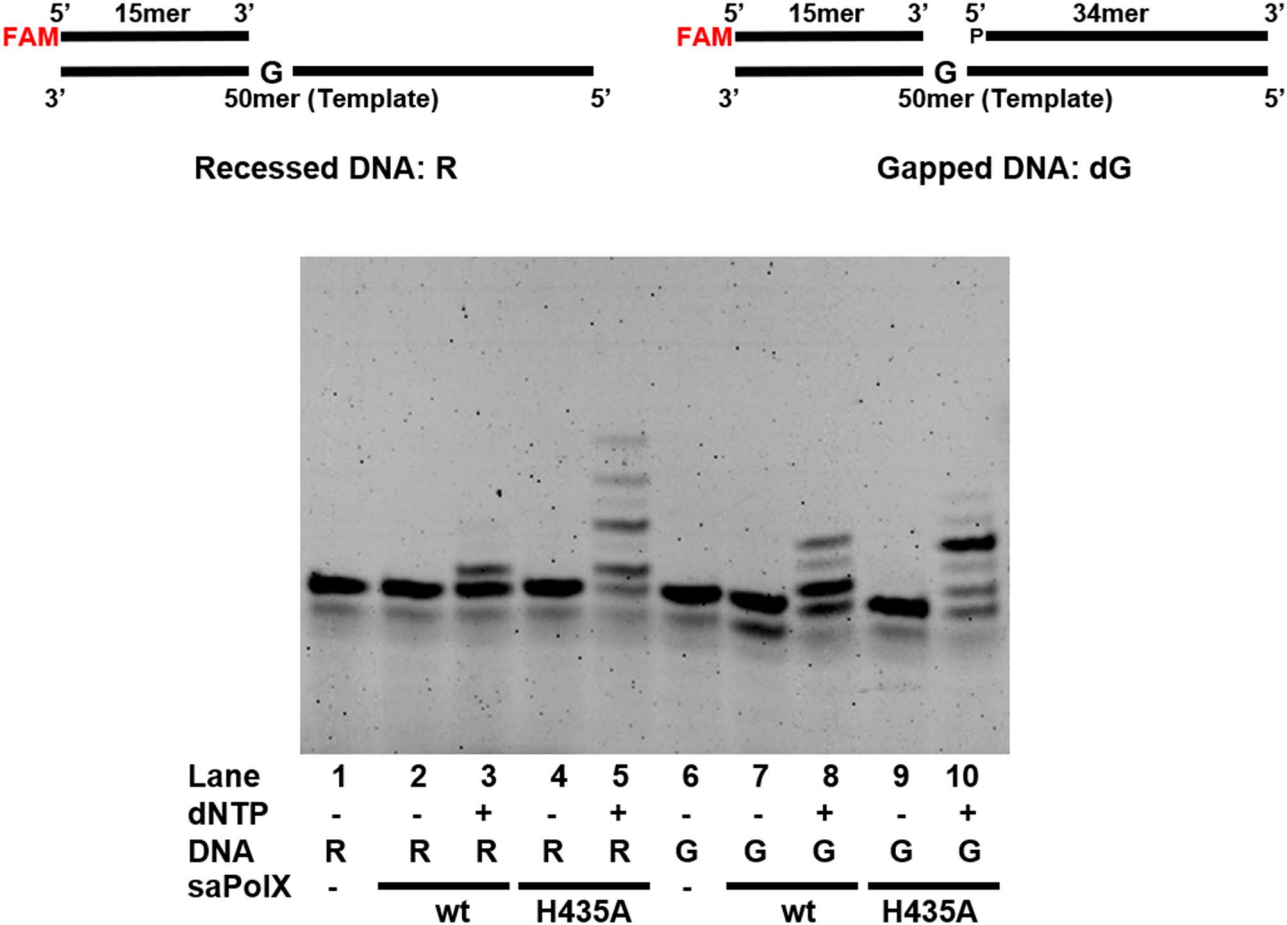
Effect of exonuclease activity on polymerase activity of saPolX. Primer extension assays were performed on a recessed (R) and gapped DNA (dG) substrates to check the role of exonuclease activity on restricting the nucleotide insertion by polymerase domain of saPolX. The enzymatic activities were checked on both the DNA substrates with wt-saPolX and H435A mutant. The results show that H435A exo- mutant inserts higher number of nucleotides on both the substrates as compared to the wt-saPolX. The exonuclease activity residing in the PHP domain of saPolX limits the incorporation of nucleotides by the polymerization activity of the polymerase domain.

We examined the polymerization activity of wt-saPolX and H435A mutant on the 1-nt gapped DNA substrate in the presence of all the nucleotides mixture (dNTPs). Both wt-saPolX and H435A mutant incorporated more than one nucleotide in the gapped substrate which suggested the displacement of the downstream strand (Fig. 5). H435A mutant showed comparatively higher downstream strand displacement than the wt-saPolX. To understand the role of exonuclease activity residing in the PHP domain in limiting the addition of nucleotides by the polymerase domain, the polymerase activity of saPolX was compared on recessed DNA substrate (Fig. 5). We found that wt-saPolX showed limited polymerization activity as compared to the H435A mutant on the recessed DNA substrate.

### Fidelity and incorporation of ribonucleotides and oxidized nucleotides

**Figure 6:**
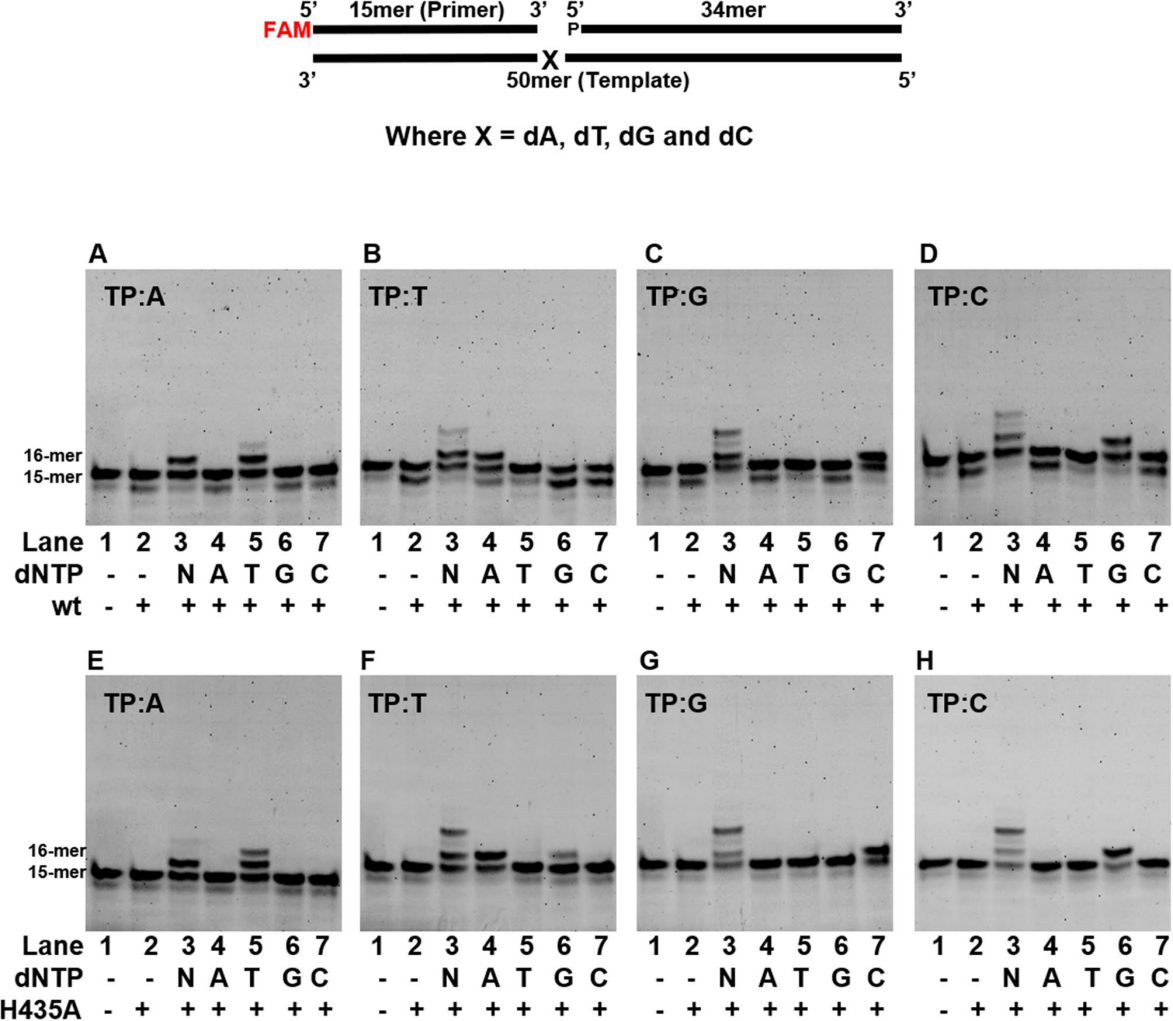
Fidelity of DNA synthesis by saPolX. Primer extension assays were conducted to assess the ability of saPolX to incorporate dATP, dTTP, dGTP and dCTP opposite the four possible template nucleotides- dA, dT, dG and dC. The identity of the template nucleotide is mentioned at the top of each gel. wt-saPolX shows incorporation according to normal Watson–Crick base pairing (**A**–**D**). H435A mutant shows accurate incorporation opposite templating bases A, G and C (**E**–**H**) but exhibits significant misincorporation of dGTP opposite template dT (**F**).

We examined the fidelity of wt-saPolX on four gapped DNA substrates wherein each substrate represents one of the four nucleotides (A, T, G or C) at the templating position. Wildtype-saPolX exhibited high fidelity on all four gapped DNA substrates (Fig. 6A–D). To understand the contribution of the PHP domain towards the replication fidelity, we checked the fidelity of the H435A mutant as well (Fig. 6E–H). We observed that the H435A mutant showed significant misincorporation of dGMP opposite templating nucleotide dT. To analyze the role of PolXc in misincorporation, we checked the catalytic efficiency for the incorporation of dGMP opposite templating nucleotide dC (k_cat_/K_M_ = 4.10 × 10^–1^ μM^−1^ min^−1^) and opposite templating nucleotide dT (k_cat_/K_M_ = 2.95 × 10^–4^ μM^−1^ min^−1^) by H435A mutant (Table 1). The misincorporation of dGMP opposite templating nucleotide dT was 1,350-fold lesser than its correct incorporation opposite templating nucleotide dC (Table 1).

**Table 1:** Steady-state kinetic parameters for nucleotide incorporation by exonuclease mutant saPolX-H435A on 1 nucleotide gapped DNA substrates.

Templating nucleotide	Incoming nucleotide	k_cat_ (min^−1^)	K_M_ (μM)	k_cat_/K_M_ (μM^−1^ min^−1^)	f_incorporation_
dA	dTTP	5.82 × 10^−2^ ± 5.3 × 10^−3^	6.38 ± 0.63	9.14 × 10^−3^	1
dT	dATP	8.07 × 10^−1^ ± 5.3 × 10^−2^	1.32 ± 0.16	0.616	1
dG	dCTP	7.68 × 10^−1^ ± 5.9 × 10^−2^	3.76 ± 0.39	0.205	1
dC	dGTP	7.68 × 10^−1^ ± 9.9 × 10^−2^	1.87 ± 0.27	0.410	1
dT	dGTP	1.37 × 10^−2^ ± 1.5 × 10^−3^	47.00 ± 7.72	2.95 × 10^−4^	4.8 × 10^−4^
dA	rUTP	ND	ND	ND	–
dT	rATP	ND	ND	ND	–
dG	rCTP	2.1 × 10^–2^ ± 3.3 × 10^–3^	137.63 ± 9.40	1.52 × 10^–4^	7.4 × 10^–4^
dC	rGTP	1.17 × 10^–2^ ± 1.9 × 10^–3^	246.50 ± 42.11	4.74 × 10^–5^	1.15 × 10^–4^
dG	rGTP	ND	ND	ND	-
dA	8oxodGTP	1.04 × 10^–2^ ± 1.5 × 10^–3^	57.35 ± 10.39	1.83 × 10^–4^	0.02
dC	8oxodGTP	ND	ND	ND	-
dT	2-OH-dATP	3.76 × 10^–2^ ± 5.0 × 10^–3^	4.46 ± 0.70	8.55 × 10^–3^	0.014
dG	2-OH-dATP	ND	ND	ND	-
dC	2-OH-dATP	ND	ND	ND	-

For each combination, the templating position nucleotide is mentioned in column 1 and the incoming nucleotide is mentioned in column 2.

Not determined is abbreviated as ND.

**Figure 7:**
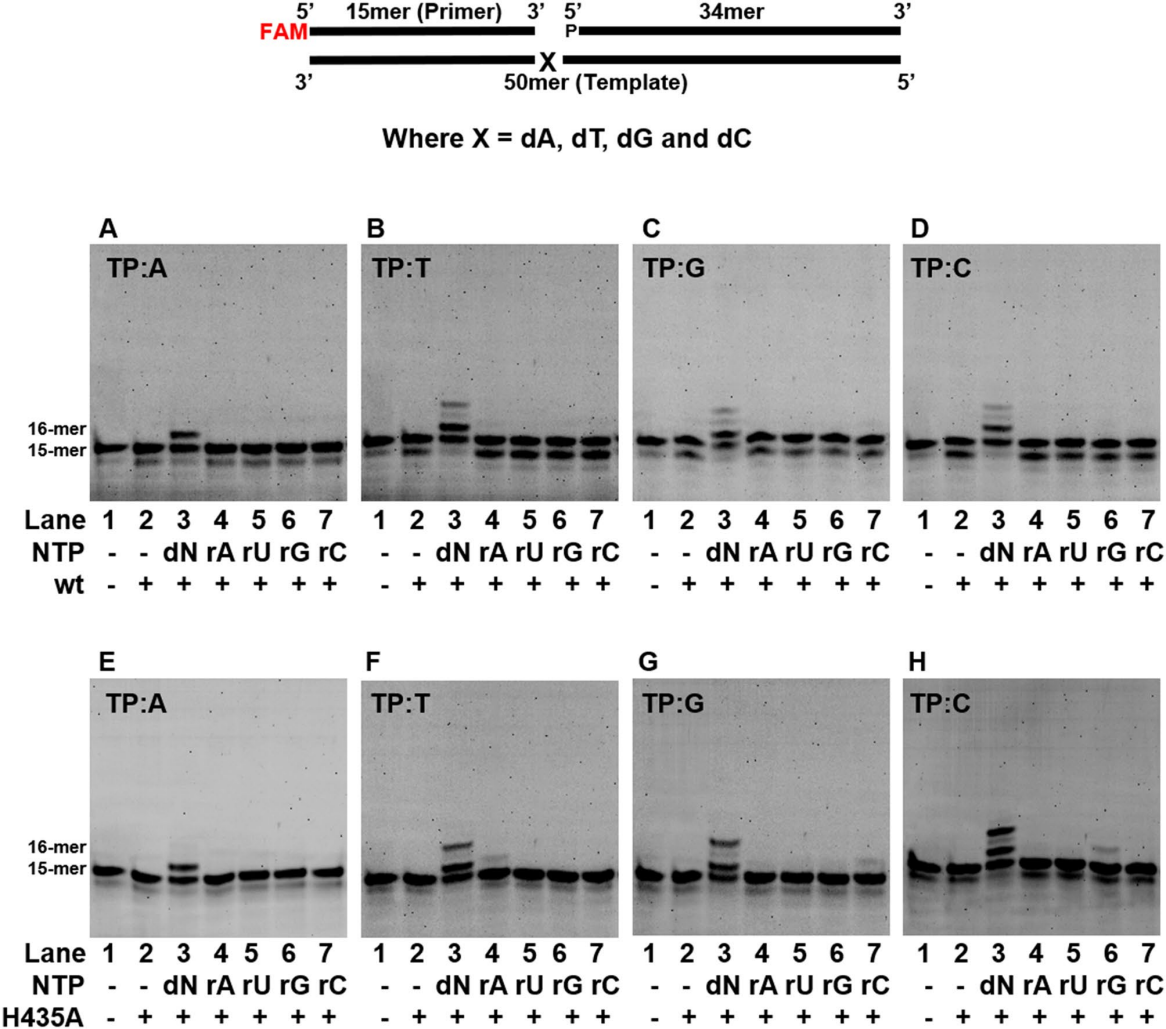
Comparison of rNTP incorporation by wt-saPolX and H435A. Primer extension assays were conducted to assess the ability of saPolX to incorporate rATP, rUTP, rGTP and rCTP opposite the four possible template nucleotides- dA, dT, dG and dC. The identity of the template nucleotide is mentioned at the top of each gel picture. wt-saPolX does not incorporate any rNTP as shown in panels (**A**–**D**), respectively. saPolX-H435A shows incorporation of (**F**) rATP opposite dT, (**G**) rCTP opposite dG and (**H**) rGTP opposite dC (**E**) except incorporation opposite dA.

Members of the X-family are known to incorporate rNTPs and hence the ability of saPolX to discriminate between dNTPs and rNTPs was evaluated. For this, the rNTP incorporation activity of wt-saPolX and H435A in the case of all the four DNA substrates was assayed. We observed that wt-saPolX was not able to incorporate any of the rNTPs (Fig. 7A–D), whereas the exonuclease mutant H435A was able to add rNTPs to various efficiencies (Fig. 7E–H). H435A mutant inserts rAMP opposite template nucleotide dT, rCMP opposite template nucleotide dG and rGMP opposite template nucleotide dC (Table 1). The incorporation of incoming rCMP against templating dG nucleotide (k_cat_/K_M_ = 1.52 × 10^–4^ μM^−1^ min^−1^) and incoming rGMP against templating dC nucleotide (k_cat_/K_M_ = 4.74 × 10^–5^ μM^−1^ min^−1^) were quantifiable but that of other ribonucleotides were not (Table 1). Overall, the experiments with wt- and exo- versions of saPolX show that the ability to prevent rNMP incorporation is enhanced by exonuclease activity residing in the PHP domain.

**Figure 8:**
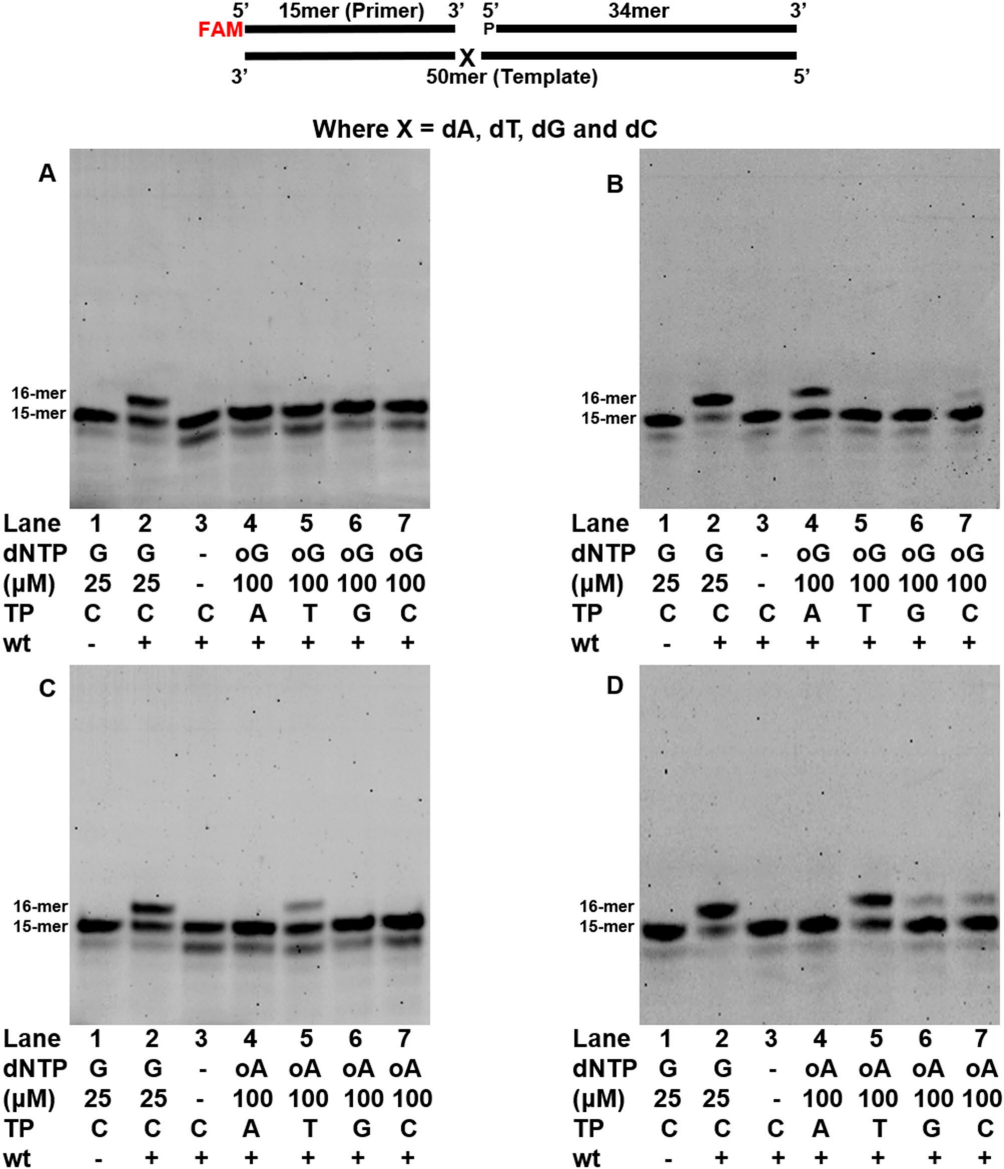
Comparison of incorporation of oxidized nucleotides by wt and mutant versions of saPolX. The incorporation of 8oxodGTP (oG) opposite all four possible templating deoxynucleotides was compared to the incorporation of dGTP opposite dC for (**A**) wt-saPolX and (**B**) mutant H435A. Whereas wt-saPolX does not incorporate 8oxodGTP at all, H435A incorporates 8oxodGTP preferentially opposite template dA instead of dC. The incorporation of oxidized nucleotide 2-OH-dATP (oA) opposite all four possible templating deoxynucleotides was compared to that of incorporation of dGTP opposite dC for (**C**) wt-saPolX and (**D**) mutant H435A. The native enzyme incorporated 2-OH-dATP opposite dTTP but the mutant version misincorporated the damaged nucleotide opposite template dG and dC also.

Many DNA polymerases misincorporate oxidized nucleotides such as 8oxodGTP opposite incorrect templating nucleotide. We examined the ability of wt-saPolX and H435A to incorporate oxidized nucleotides on all the four gapped DNA substrates in the presence of Mg^2+^. wt-saPolX was unable to incorporate 8oxodGMP opposite any of the templating nucleotides (Fig. 8A). However, the saPolX- H435A was able to insert 8oxodGMP against templating nucleotide dA and dC both with a clear preference for templating nucleotide dA (Fig. 8B). The steady-state kinetics data supported this observation where we could find the catalytic efficiency for the incorporation of 8oxodGMP opposite templating nucleotide dA (k_cat_/K_M_ = 1.83 × 10^–4^ μM^−1^ min^−1^) by H435A mutant (Table 1). wt-saPolX was able to insert 2-OH-dAMP against templating nucleotide dT in gapped DNA substrate to a significant degree (Fig. 8C), whereas H435A was able to misincorporate 2-OH-dAMP against templating nucleotides dG and dC as well (Fig. 8D) (Table 1).

### Exonuclease activity of saPolX and H435A mutant

**Figure 9:**
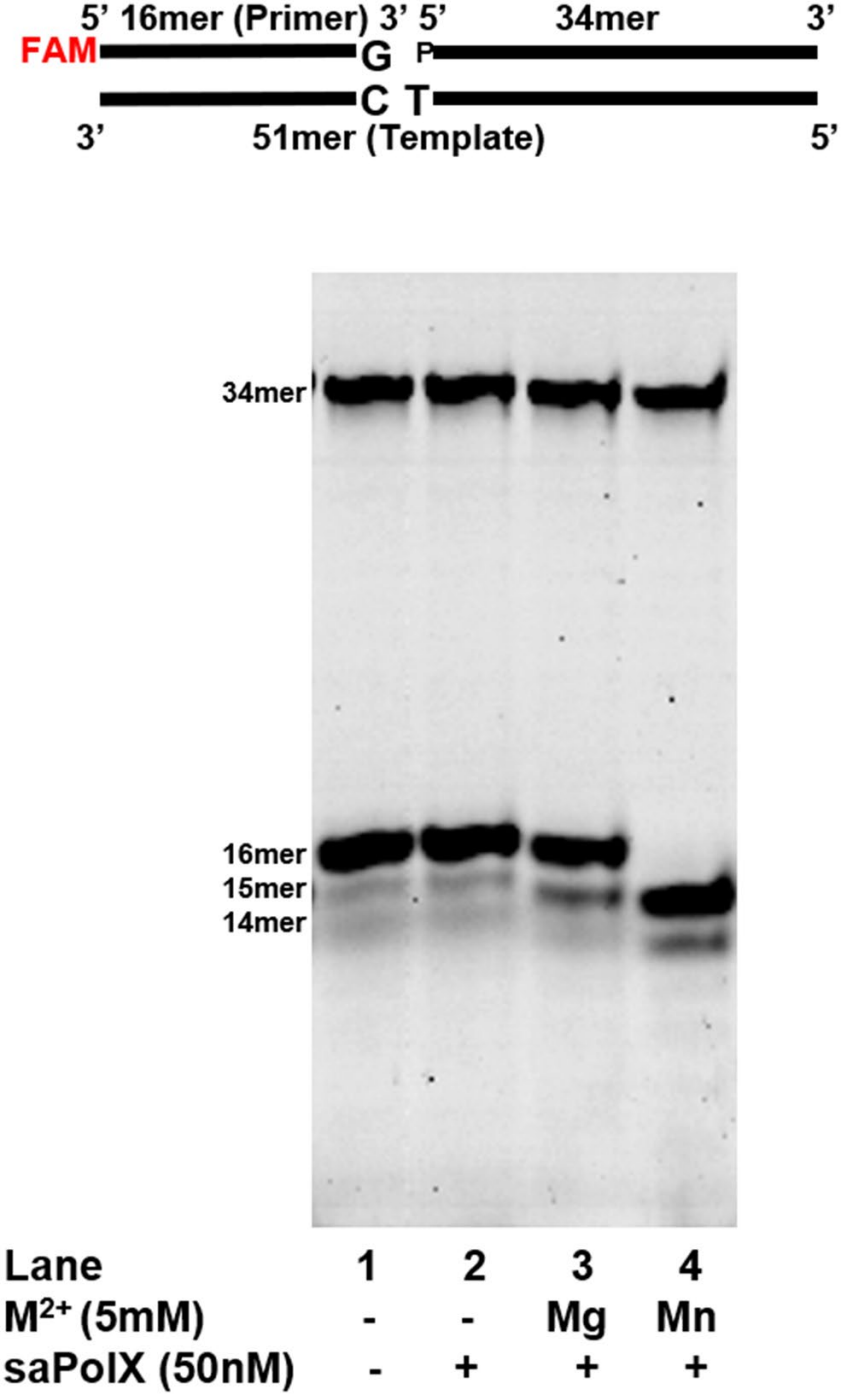
Metal ions required for optimal exonuclease activity of saPolX. The exonuclease activity was tested in the presence of Mg^2+^ and Mn^2+^ metal ions for wt-saPolX which was treated with 0.2 M EDTA during purification. The gapped DNA substrate (C:G) is displayed at the top of the gel. LC represents the loading control (6FAM labelled 34mer DNA oligonucleotide) added in each lane for quantification. The result shows that wt-saPolX degrades ~ 20% and 100% substrate in the presence of Mg^2+^ and Mn^2+^ metal ions, respectively. The experiments show that wt-saPolX prefers Mn^2+^ metal ion to exhibit exonuclease activity.

wt-saPolX exhibited 3′–5′ exonuclease activity on gapped DNA substrates (Fig. 4). We checked the exonuclease activity of saPolX on 1 nucleotide gap DNA substrate in the presence of biologically relevant divalent cations Mg^2+^ and Mn^2+^. Wildtype saPolX showed exonuclease activity even in the absence of exogenously added divalent metal ion. This was hypothesized to happen due to the presence of the prebound metal ion in the protein. It was imperative to remove this prebound metal ion to know with certainty which metal ion can be utilized by saPolX for exonuclease activity. To remove the prebound metal ion, we purified the wt-saPolX protein in the presence of 0.2 M EDTA as described in the methods section. The protein purified with this strategy showed negligible exonuclease activity in the absence of externally added divalent metal ions (Fig. 9). Our results showed that DNA substrate was completely degraded in the presence of the Mn^2+^ metal ion (Fig. 9). Surprisingly, we observed approximately 20% degradation of the DNA substrate in the presence of Mg^2+^ metal ion as well. This was an unexpected result because all the other reported bacterial PolXs show no exonuclease activity in the presence of Mg^2+^. These results motivated us to check the metal ion concentration-dependent exonuclease activity of wt-saPolX on gapped DNA substrate (Supplementary Fig. S2). We found that wt-saPolX showed maximum degradation activity with Mn^2+^ even at 0.5 mM concentration. In the presence of Mg^2+^, wt-saPolX showed maximum 34% exonuclease activity at 4 mM metal ion concentration which reduced as the metal ion concentration was increased. This confirmed that Mn^2+^ metal ion was the preferred ion for exonuclease activity but PolX can utilize Mg^2+^ for exhibiting exonuclease activity. Thus, we concluded that different dPols from the X family may exhibit different preferences for metal ion cofactors.

### Time-course analysis of the exonuclease activity

**Figure 10:**
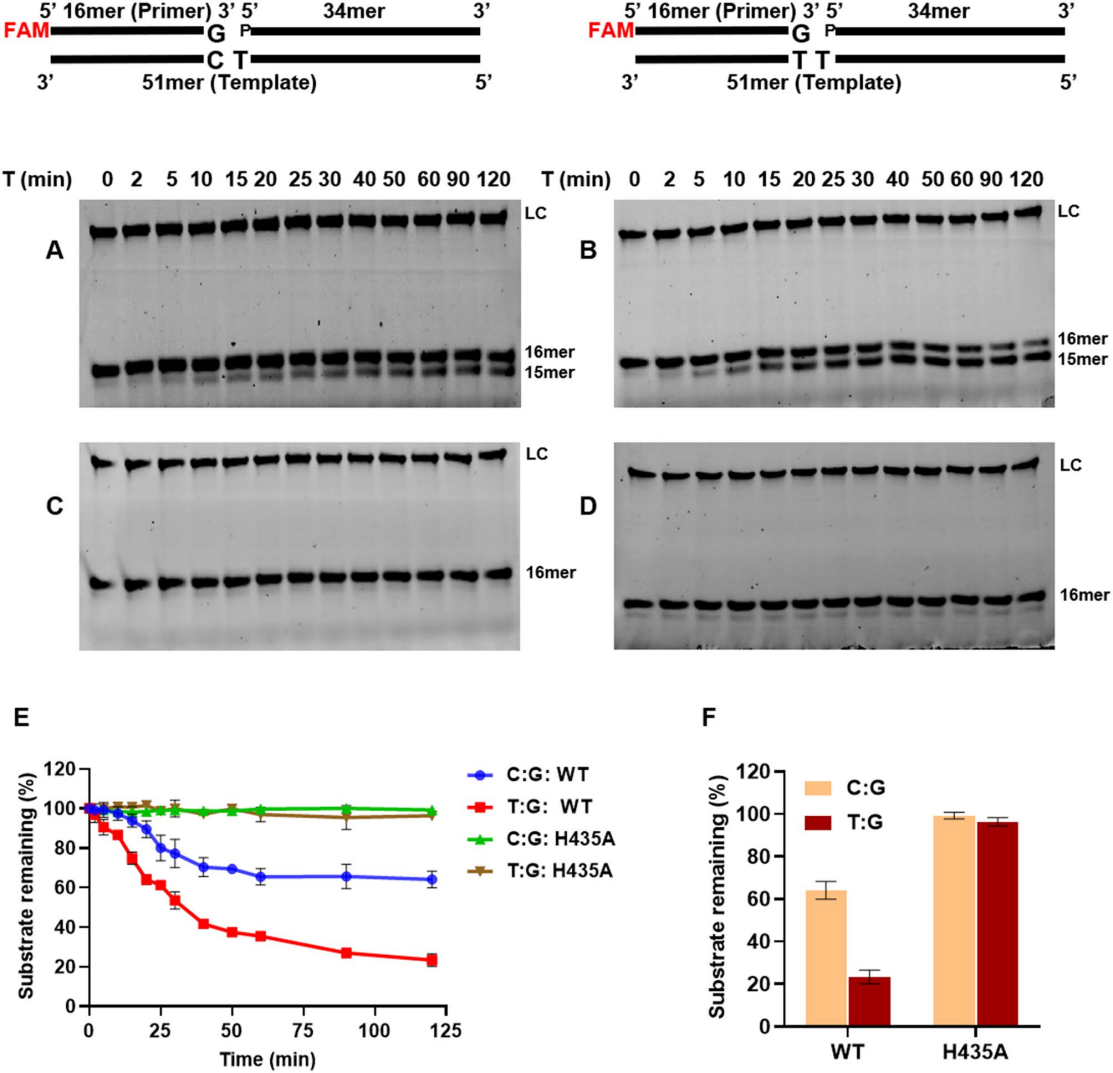
Proofreading activity of wt-saPolX. Time-course analysis of 3′–5′ exonuclease activity exhibited on 1 nucleotide gapped DNA substrates bearing normal (C:G) and mismatched (T:G) base pairs in the presence of Mn^2+^ by saPolX wt (12.5 nM) (**A**,**B**) and H435A (12.5 nM) (**C**,**D**), respectively. LC represents the loading control (6FAM labelled 34mer DNA oligonucleotide) added in each lane for quantification. The time course of exonuclease activity of wt-saPolX and H435A on matched and mismatched gapped DNA are displayed (**E**). The exonuclease activity of wt-saPolX and H435A at 120 min incubation was also compared (**F**). saPolX showed higher exonuclease activity when it encountered a mismatched base pair as compared to the correctly paired Watson–Crick base pair.

We observed that wt-saPolX showed high fidelity, and H435A was able to misincorporate dGMP opposite templating dT nucleotide in the gapped DNA substrate. Thus the exonuclease activity was compared between wt-saPolX and H435A on two gapped DNA substrates representing typical Watson–Crick paired substrate (C:G) and mismatched DNA substrate (T:G). Time-course analysis of the exonuclease activity (Fig. 10) showed that wt-saPolX was able to degrade both correctly paired DNA substrate and mismatched DNA substrate but the activity on the mismatched gapped DNA substrate was 2.7-fold higher than the correctly paired substrate (Fig. 10F). As expected saPolX-H435A showed no activity on any of the substrates.

**Figure 11:**
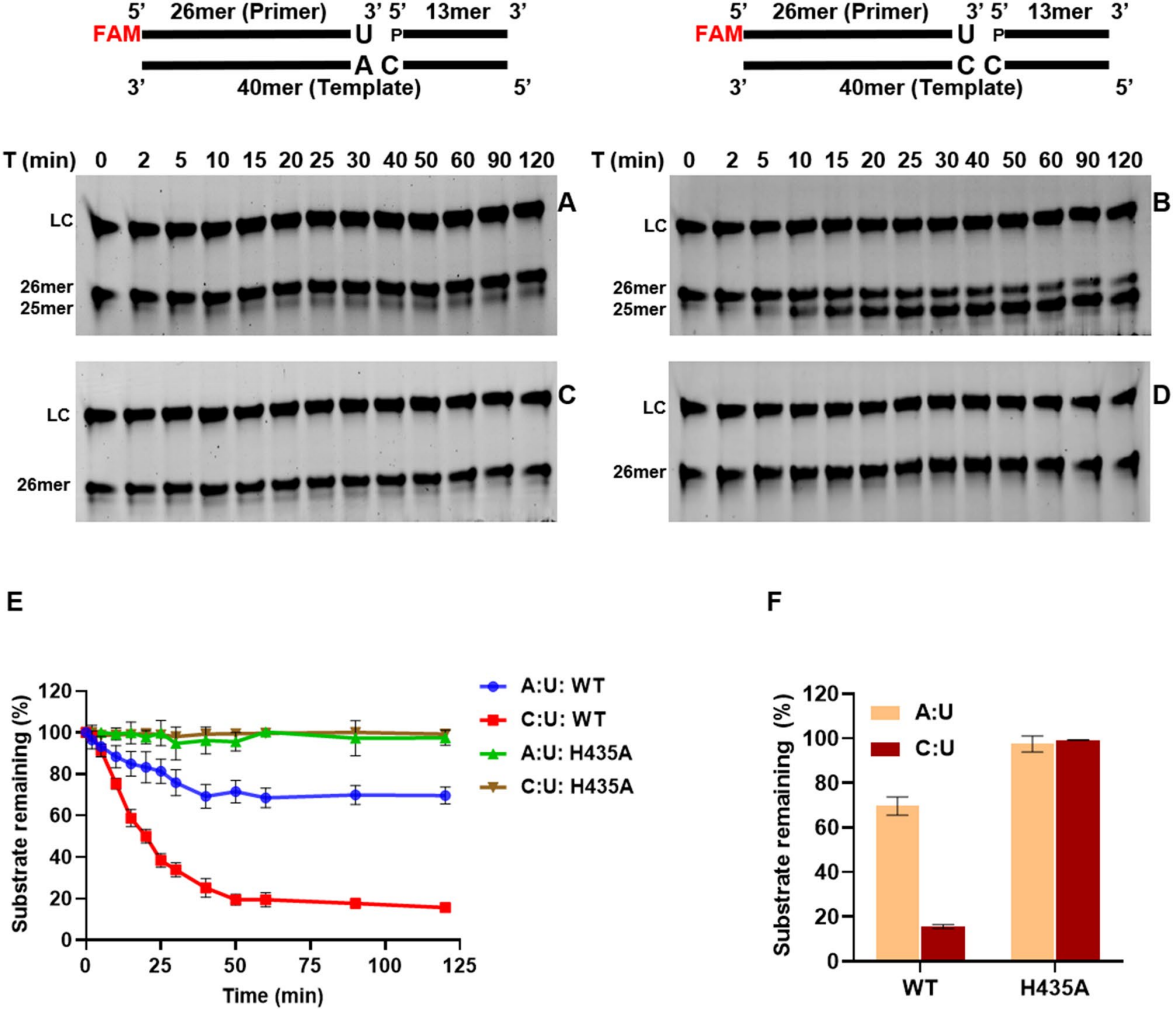
Exonuclease activity of wt-saPolX to remove ribonucleotides. Time-dependent 3′–5′ exonuclease activity exhibited on gapped DNA containing rUMP incorporated against templating A and C nucleotide in the presence of divalent metal ion Mn^2+^ by wt-saPolX (**A**,**B**) and H435A mutant (50 nM each) (**C**,**D**), respectively. LC represents the loading control added in each lane for quantification. In graph (**E**), the time course analysis of wt-saPolX and H435A for ribonucleotide excision is displayed. In bar graph (**F**), the level of ribonucleotide excision by wt-saPolX and H435A for 120 min incubation is compared.

**Figure 12:**
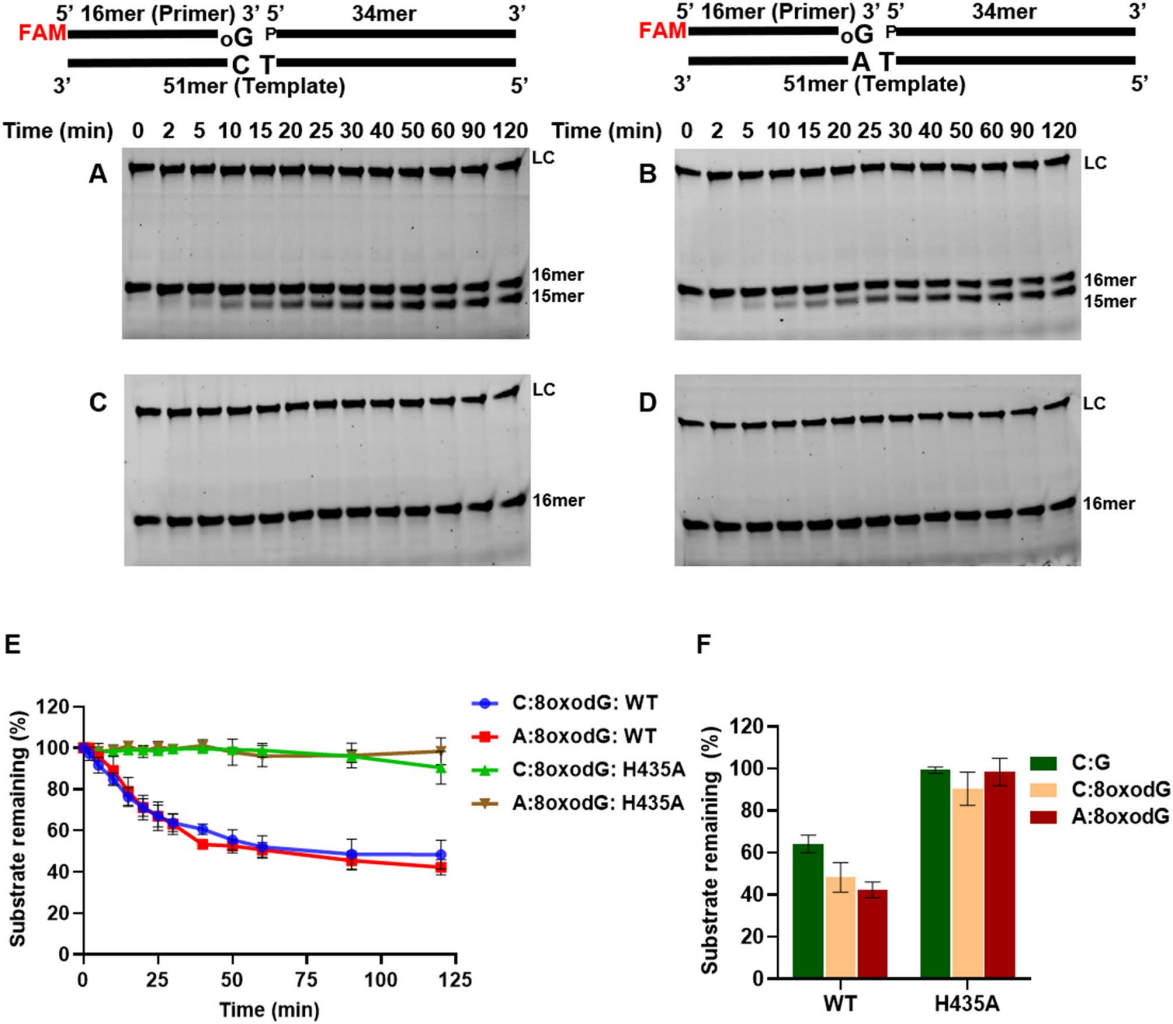
Exonuclease activity of wt-saPolX to remove 8oxodGMP present at primer terminus. Time-course analysis of exonuclease activity exhibited on gapped DNA bearing 8oxodG incorporated opposite templating C (C:8oxodG) and A (A:8oxodG) nucleotides in the presence of Mn^2+^ by wt-saPolX (12.5 nM) (**A**,**B**) and H435A mutant (12.5 nM) (**C**,**D**), respectively, are displayed. LC represents the loading control added in each lane for quantification. The decrease in the levels of the substrates bearing 8oxodGMP at the 3′ end in the presence of Mn^2+^ is shown in graph (**E**). The amount of substrate remaining after 120 min of incubation for DNA substrates C:8oxodG, A:8oxodG and C:G are compared (bar graph **F**). These experiments show that wt-saPolX can excise out mis-incorporated oxidized nucleotides in the presence of Mn^2+^ ions.

We performed the time-course analysis of exonuclease activity with wt-saPolX and H435A on gapped DNA substrates containing rUMP incorporated against templating nucleotide dA (A:U) and dC (C:U) (Fig. 11). Our results showed that H435A was unable to remove incorporated rUMP from either of the substrates but wt-saPolX was able to degrade both substrates. We noticed that wt-saPolX was able to remove rUMP with greater efficiency for the mismatched pair (rU:dC) (4.5-fold higher) than for a Watson–Crick pair (rU:dA) (Fig. 11F). Overall, the PHP domain in the presence of Mn^2+^ ions significantly enhances the ability of saPolX to prevent ribonucleotide incorporation.

wt-saPolX did not exhibit any incorporation of 8oxodGMP but H435A was able to incorporate 8oxodGMP against templating nucleotide dA preferred over dC. Time-course analysis of exonuclease activity of wt-saPolX on substrates containing 8oxodGMP incorporated against templating nucleotide dC (C:8oxodG) and dA (A:8oxodG) showed that wt-saPolX was able to remove 8oxodGMP with approximately equal efficiency (Fig. 12). As expected, H435A was not able to remove the 8oxodGMP from any of the substrates. H435A showed no exonuclease activity on any of the DNA substrates which confirms that exonuclease activity resides in the PHP domain.

### Polymerase activity on mismatched DNA substrate

**Figure 13:**
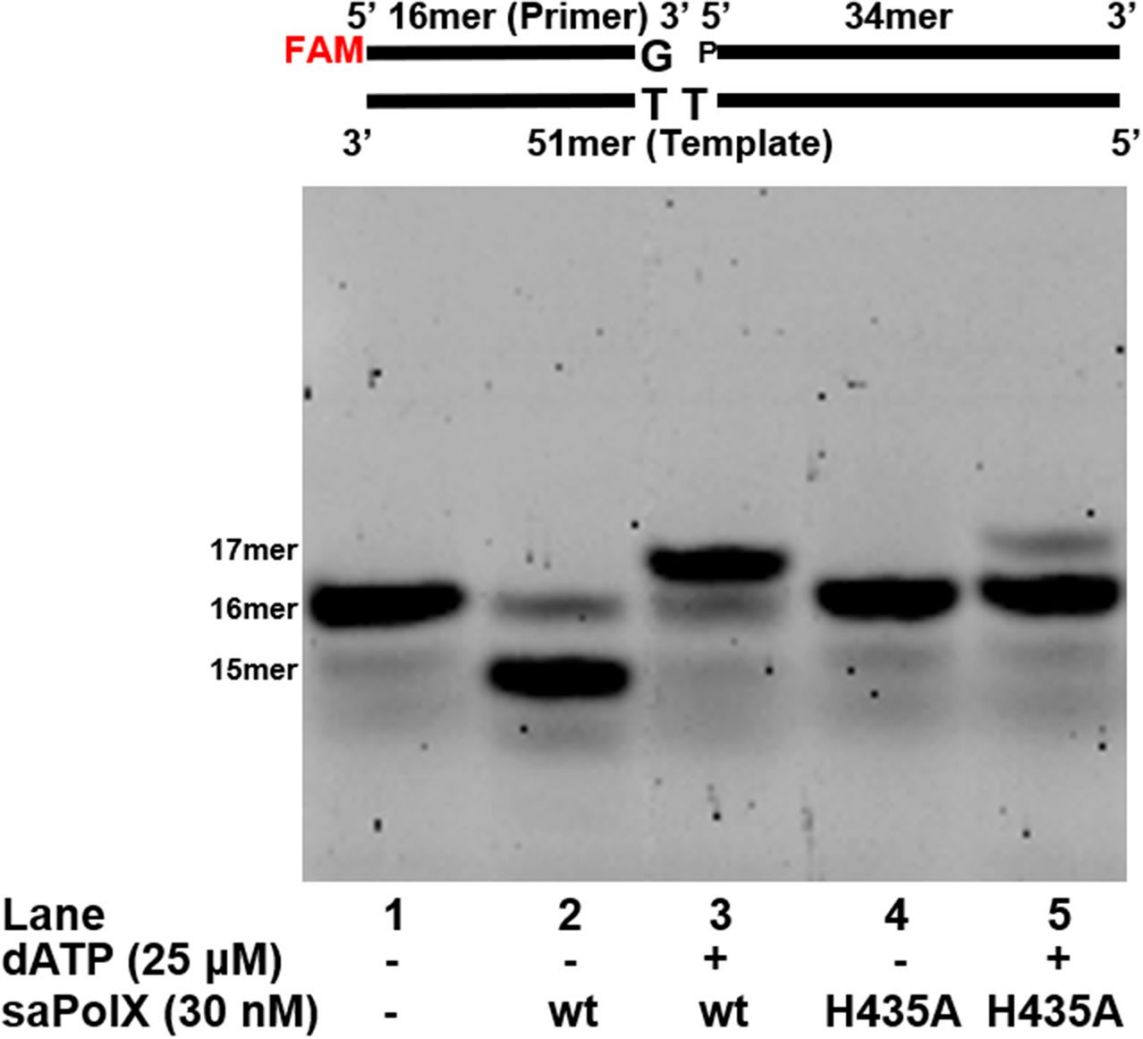
Polymerase activity on mismatched base pair by saPolX. The polymerase activity of wt and H435A exo- mutant were tested on the mismatched gapped DNA substrate. The mismatched gapped DNA substrate (T:G) is displayed at the top of the gel. The result shows that wt-saPolX removes the mismatched base dGMP paired opposite templating base dT and then extends it efficiently by incorporating dATP. H435A mutant does not remove the mismatched base pair and shows less incorporation of dATP. The experiment shows that exonuclease activity residing in the PHP domain of saPolX proofreads the misincorporated base pair which is extended by the polymerase domain of saPolX.

We found that wt-saPolX showed high fidelity, and exhibited preferential excision of mismatched base pairs. To understand the contribution of exonuclease activity residing in PHP domain towards the proofreading activity of saPolX, we checked polymerase activity of saPolX on gapped mismatched DNA substrate (T:G) (Fig. 13). Results showed that exonuclease proficient wt-saPolX removed the misincorporated base pair and then efficiently extended it by correctly adding dAMP. The exonuclease deficient H435A mutant was unable to degrade the mismatched base pair and extended the mismatched primer terminal inefficiently. The results show that the saPolX exhibits proofreading.

In summary, our experiments show that the exonuclease activity present in the PHP domain can remove misincorporated deoxyribo-, ribo- and oxidized nucleotides and thus contribute substantially to the fidelity of DNA synthesis by saPolX.

## Discussion

The polymerase assays indicate that saPolX is an accurate polymerase like previously characterized X-family dPols. The kinetics experiments for H435A indicate that saPolXc incorporates correct nucleotides preferentially. It was seen that the catalytic efficiency of incorporation of dTMP opposite dA is less than for the other Watson–Crick incipient base pairs though the structural basis for this asymmetric activity is not known. ttPolX also shows asymmetric incorporation of dNTPs by incorporating purines favourably over the pyrimidines. The structural explanation for this biased activity in ttPolX may be due to a conserved residue Ser266 in *Thermale* order^63^. On mutating Ser266 into Asn, which is conserved in other prokaryotic PolXs including saPolX, the catalytic efficiency for all the four dNTPs was nearly equal in ttPolX. Overall, from our kinetics and time course experiments, it appears that fidelity of saPolXc is inherently high which is significantly increased due to the exonuclease activity present in the PHP domain. bsPolX and ttPolX both have been shown to synthesize DNA faithfully in the presence of Mg^2+^ metal ion^5,6^. It should be mentioned here that both bsPolX and ttPolX^6,26^, exhibit no 3′–5′ exonuclease activity in the presence of Mg^2+^. In comparison, saPolX exhibits significant Mg^2+^ dependent exonuclease activity and this will ensure that there is minimal loss of fidelity of DNA synthesis even in a Mn^2+^ deficient scenario.

The incorporation of rNMPs in the DNA leads to the local structural alterations from B form to A form^72^ and also, renders DNA more vulnerable to the cleavage due to the higher chemical reactivity of the 2′-OH group under the cellular conditions^73^. These structural and chemical changes introduced by incorporated rNMPs prevents the formation of nucleosomes^74^ and also leads to the genomic instability^75^ which causes cell death^76^. In the case of bacterial X family polymerase, ttPolX shows an inefficient rNTPs incorporation as compared to dNTPs which have been solely attributed to the lower binding affinity for rNTPs as compared to dNTPs^66^. In the case of saPolX, polymerase activity can incorporate rNTPs especially opposite template dG and dC. Although the catalytic efficiencies of rNTP incorporation are low, this activity may be physiologically relevant as the cellular concentration of rNTPS is always higher than dNTPs. The time-dependent exonuclease activity studies show that wildtype saPolX removes the incorporated rNTPs efficiently. Overall, these results indicate that the PHP domain contributes substantially to the ability of saPolX to prevent ribonucleotide incorporation.

saPolXc exhibits significant ability to misincorporate 8oxodGMP opposite dA. This damaged nucleotide arises due to oxidation of the nucleotide pool by reactive oxygen species (ROS)^50^. 8oxodGMP can either correctly pair with cytosine in its anti-conformation using Watson–Crick pairing rules (error-free) or can incorrectly make Hoogsteen base pair with adenosine in its syn conformation (error-prone)^54^. 2-hydroxy-2′-deoxyadenosine triphosphate (2-OH-dATP), generated by the oxidation of dATP, is another prominent lesion which has been shown to be misincorporated by DNA polymerases opposite guanine in template DNA during DNA replication, causes spontaneous mutagenesis^52^. The misincorporation of 8oxodGMP and 2-OH-dAMP by DNA polymerases frequently leads to A:T → C:G and G:C → T:A transversions, respectively. The repair polymerases from X family like human Polβ and polλ, which are involved in DNA repair pathways like BER and NHEJ, show a strong ability to incorporate 8oxodGMP opposite dA^56,58,61^. Among bacterial X family polymerases, ttPolX incorporates 8oxodGMP preferentially opposite adenosine as compared to 8oxodGMP opposite cytosine, but its efficiencies for the incorporation of 8oxodGMP are very low compared to the other undamaged dNMPs^58,63^. It is believed that the presence of Ser266 prevents the formation of the stable dA:8oxodGMP Hoogsteen base pair in the dPol active site^63^. In the case of bsPolX, there is an Asn residue present at 263 position equivalent to Ser266 of ttPolX which may form interactions with the oxygen atom at the C8 position and thus stabilizes the dA:8oxodGMP Hoogsteen base pair in the dPol active site^64^. bsPolX which is able to stabilize both anti and syn conformation of 8oxodGMP^64^ incorporates it opposite cytosine and adenosine with similar catalytic efficiencies. However, even though saPolX shows the presence of Asn residue like bsPolX, there is no detectable incorporation of 8oxodGMP opposite template dC. The saPolXc region exhibits substantial 8oxodGMP incorporation activity and the observed frequency of incorporation is 0.02. This value is substantially higher than that observed (0.005) for the error-prone DNA Polymerase IV (Y-family) from *E. coli*^46^. However, our studies show that the exonuclease activity resident in the PHP domain can remove 8oxodGMP or 2-OH-dAMP present at the 3′ end of the primer and prevent mutagenic DNA synthesis. The PHP domain of bsPolX can also excise out 8oxodGMP from the primer terminus and therefore the ability to remove misincorporated oxidized nucleotides may be a general property of many prokaryotic X-family DNA polymerases^64^.

**Figure 14:**
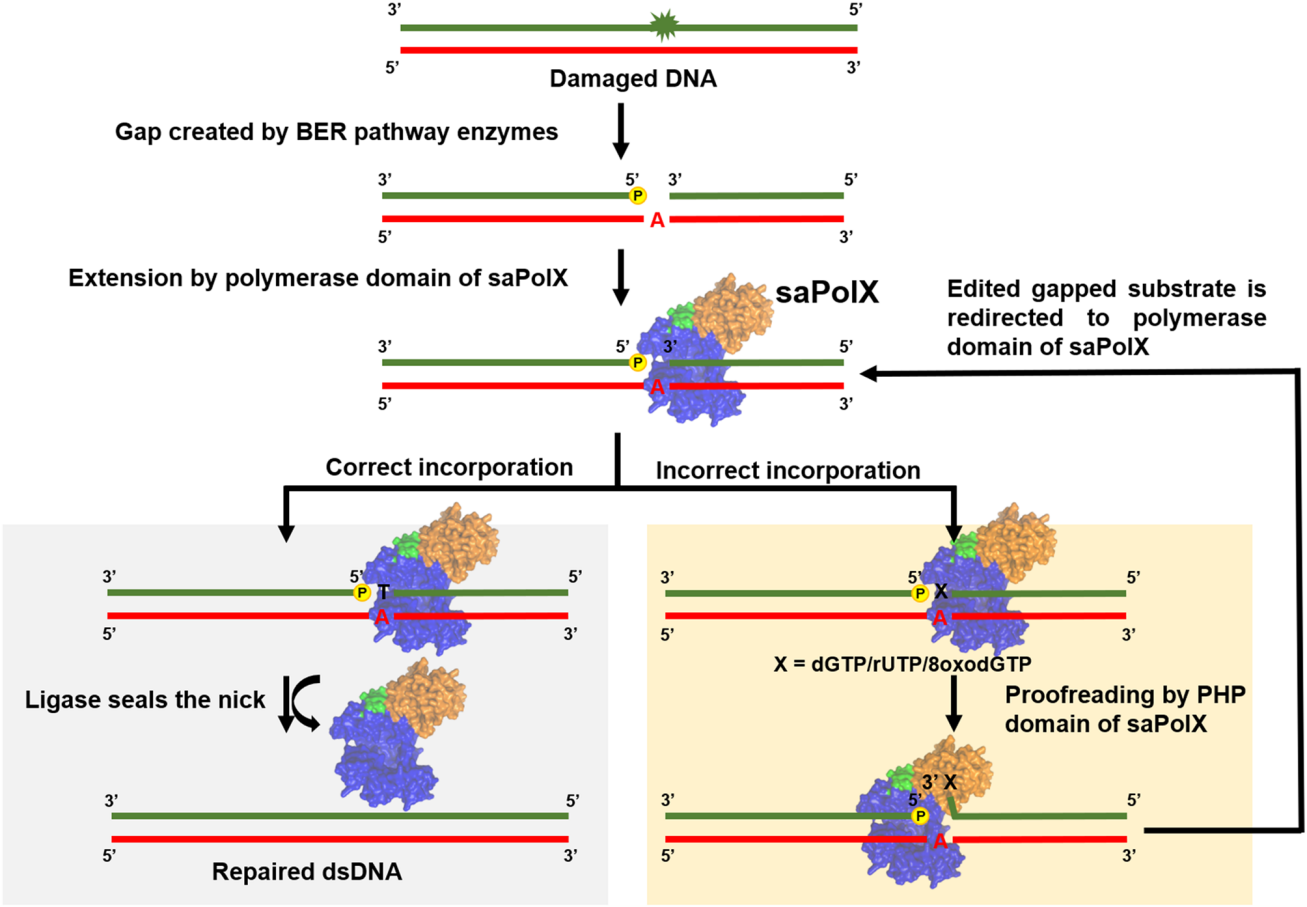
Model for the high-fidelity DNA synthesis by saPolX. The core polymerase domain, PHP domain and linker connecting these two domains are displayed in blue, orange and green colors, respectively. The schematic shows the interplay between the 5′–3′ polymerization and 3′–5′ exonuclease activities residing in polymerase and PHP domains, respectively. saPolXc incorporates either correctly paired nucleotide or mismatched nucleotide on gapped DNA substrate generated during BER pathway. The correctly filled DNA substrate is sealed by the ligase enzyme in the subsequent step. The DNA substrates with misincorporated deoxyribo-, ribo- and oxidized nucleotides are directed towards the PHP domain. The PHP domain removes these bases and redirects the gapped DNA substrate to the polymerase domain to repair it correctly.

In conclusion, our results prove that exonuclease activity present in the PHP domain interacts with the PolXc domain and scans for the presence of incorporation of the wrongly matched base, wrong sugars containing nucleotide and oxidized nucleotide (summarized in Fig. 14). The inherent high fidelity of the polymerase activity regarding dNMP incorporation combined with the ability of the exonuclease domain of saPolX to remove misincorporated erroneous dNMPs, rNMPs or oxidized nucleotides ensures that DNA synthesis by this enzyme is accurate. Our studies reinforce the fact that the PHP domain is essential for proofreading activity in the prokaryotic bacteria. Inhibition of the activity of the PHP domain may lead to significant enhancement in the mutation and the incorporation of rNMPs and oxidized nucleotides in the genome. Since all these events adversely affect the survival of the organism, the PHP domain may be an attractive target for the therapeutic interventions and inhibitors of this activity may act as powerful adjuvants for existing drugs against *S. aureus*.

## Methods

### Materials

The *S. aureus* subsp. *aureus* COL (GenBank: CP000046.1) genomic DNA was a generous gift from Dr. Deepti Jain’s laboratory at RCB. The DNA altering enzymes, including restriction enzymes, T4 DNA Ligase and Phusion Polymerase were bought from NEW ENGLAND BioLabs.

The QuikChange Lightning Multi Site-Directed Mutagenesis Kit (Agilent Technologies) was used to generate the mutants. The deoxyribonucleotide triphosphates (dNTPs), oxidized deoxyribonucleotides triphosphates and ribonucleotide triphosphates (rNTPs) were purchased from GE Healthcare. Trypsin enzyme used for limited proteolysis was purchased from Sigma-Aldrich. The DNA oligomers were purchased from Eurofins Scientific. The custom-designed fluorescent-labelled, phosphorylated and other modifications containing oligonucleotides were purchased from Keck Centre at Yale University. All other chemical reagents used were of the high grade and were purchased from commercially available sources.

### Cloning and overexpression

Using the genomic sequence information, the *SACOL1153* gene was amplified by PCR from the genomic DNA of *S. aureus* subsp. *aureus* COL strain and was cloned into the pDJN1 (modified pET-22b(+)) expression vector between BamHI and XhoI restriction sites. The PCR amplification was done using the commercially synthesized forward and reverse oligonucleotide primers 5′-GGAAAAGGATCCATGACAAAAAAAGATGTTATC-3′ and 5′-GGAAACTCGAGCTATTTCTTAAGTTTTATATTATTTTC-3′, respectively. The amplified product was purified and ligated into the pDJN1 vector that would render a fusion polypeptide with a 6x His tag at the N-terminus connected to the *SACOL1153* gene by a linker with a PreScission protease site. Point mutants of saPolX were created using QuikChange Lightning Multi Site-Directed Mutagenesis Kit (Agilent Technologies). Primers were designed for creating the catalytic mutant for polymerase activity (D193A): 5′-CGT TTT AAA GAA ATG AGC AAA GCT TTA GAT TTC ATA ATA AGT ACC-3′ and 5′-GGT ACT TAT TAT GAA ATC TAA AGC TTT GCT CAT TTC TTT AAA ACG-3′ and exonuclease activity (H435A): 5′-GAT TAT GTA ATT GGA GCT ATT GCT CAA AGC TTT AAC CAA TCA G-3′ and 5′-CTG ATT GGT TAA AGC TTT GAG CAA TAG CTC CAA TTA CAT AAT C-3′.

### Protein purification

The 6x His tag fused saPolX protein was expressed in *E. coli* BL21-CodonPlus cells. Transformed cells were cultured in 5 l of Luria Bertini (LB) broth containing 100 µg/ml Ampicillin at 37 °C at 180 RPM. The cells were grown until the optical density at wavelength 600 nm reached the value of 0.6–0.8. The cell culture was then induced using isopropyl 1-thio-β-D-galactopyranoside (IPTG) at the final concentration of 0.25 mM and was incubated for 18 h at 18 °C. Cells were harvested by centrifugation, suspended in lysis buffer with composition: 25 mM Tris–Cl buffer pH 8.0 (4 °C), 500 mM NaCl, 5% Glycerol, 5 mM β-Mercaptoethanol (βME), 0.01% IGEPAL CA-630 and 1 mM phenylmethylsulfonyl fluoride (PMSF), and then stored at -80 °C.

The frozen cells were thawed and lysed by sonication. The lysate was clarified by centrifugation at 38,900 g for 60 min at 4 °C and the resulting supernatant was filtered and loaded on to GE Healthcare HisTrap FF (5 ml) prepacked column pre-equilibrated with buffer A (25 mM Tris–Cl buffer pH 8.0, 500 mM NaCl, 5% Glycerol, 5 mM Imidazole, and 5 mM βME). The column was then washed with 120 ml of buffer A to remove non-specifically bound proteins. The bound proteins were eluted with a linear gradient of buffer A varying in Imidazole from 5 mM to 1 M. The fractions containing saPolX were confirmed using SDS PAGE and were then diluted using buffer B (25 mM Tris–Cl buffer pH 8.0, 5% Glycerol, and 5 mM βME) such that final concentration of NaCl in the protein solution was 50 mM. This diluted protein was then loaded for 12 h onto the prepacked GE Healthcare HiTrap Q-HP (5 ml) anion exchange column pre-equilibrated with buffer B. The protein was eluted using a linear gradient of buffer B varying in NaCl between 50 mM and 1 M. saPolX eluted at buffer corresponding to NaCl concentration of 260–310 mM. saPolX protein eluted was concentrated and further purified by size exclusion chromatography using a GE Healthcare HiLoad 16/600 Superdex 200 pg column equilibrated with buffer C (25 mM Tris–Cl, pH 8.0, 500 mM NaCl, 5% Glycerol and 2 mM DTT). The fractions corresponding to the major peaks were subjected to SDS-PAGE analysis and the fractions containing purified saPolX were concentrated to 50 mg/ml, aliquoted and stored at -80 °C. All the mutants of saPolX were purified using the same protocol.

To check which metal ion is utilized for exonuclease activity, the protein was purified with a slightly different protocol to remove the prebound divalent metal ion. After elution from Q-HP anion exchange column, the eluted protein fractions were diluted using buffer D (25 mM Tris–Cl buffer pH 8.0, 5% Glycerol, 5 mM βME, 50 mM NaCl and 0.2 M EDTA). The diluted protein was concentrated to ~ 2 ml and subjected to the size exclusion chromatography using Buffer C as mentioned previously.

### Limited proteolysis and peptide mass fingerprinting

For Peptide Mass Fingerprinting, 5 µg of protein was diluted in 100 mM Ammonium bicarbonate (ABC) and was incubated with 1 µg of trypsin for 12 h. The digested protein solution was mixed with α-Cyano-4-hydroxycinnamic acid (CHCA) in 1: 3 ratios respectively and was spotted on the Opti-TOF 384-Well Insert (123 × 81 mm) plate. To further remove the salt, on-plate washing of the spotted protein was performed using MS grade water. The spot was dried and another 1 μl of the matrix was added into it and then the spot on the plate was subjected to MALDI in AB SCIEX TOF/TOF 5800 instrument. The data were acquired and were analyzed using ProteinPilot software using the Mascot method algorithm in the NCBI database. ESI–MS for wt-saPolX and H435A was carried out at the Advanced Technology Platform Centre (ATPC) using a protocol published previously^77^.

### DNA polymerase assays

Primer extension assay was carried out to check the polymerase activity of purified recombinant protein saPolX. The oligonucleotides with a fluorescent label are considered as a safe substitute to radiolabeled oligonucleotides and thus fluorescently labelled oligos were used in this study. For this assay, different DNA oligonucleotide templates (Tn) (50mer) were annealed to a short primer (5′-end 6FAM labelled) (P1) at their 3′ end and with another primer (containing phosphate at 5′end) (P2) at their 5′ end to create the 1 nucleotide gapped DNA substrates. The different oligonucleotides used in this study are listed in Table 2. The recessed DNA substrate R which represents the 5′ overhang was prepared by annealing template oligonucleotide T3 with primer P1 in ratio 1: 1.5. Different gapped substrates were constituted by annealing the different templates and primers P1 and P2 in the ratio 1: 1.5: 2.25 (T: P1: P2). The constituted DNA substrates are listed in Table 3. For checking the polymerase activity, the reaction mixture (20 μl) comprised of 25 mM HEPES pH 7.5, 25 μM of all or one dNTP, 50 nM of DNA substrate, 5 mM MgCl_2_, 0.05 mg/ml BSA, 1 mM of DTT and 30 nM of saPolX (wild type or H435A mutant) was incubated at 37 °C for 120 min. To check the fidelity of the polymerase, reactions were carried out with a single base dNTP at a time, incubated for each 1 nucleotide gapped substrate presenting one type of base at the templating position. If the enzyme is functional, it will extend the 3′-OH of the primer by adding supplied nucleotide (dNTP/rNTP/oxidized nucleotides) opposite to the template. The concentration used for rNTPs and oxidized nucleotides (8oxodGTP and 2-OH-dATP) was 100 μM. After incubation for 2 h, the reactions were terminated by addition of the 10 *μ*L of stop solution (80% formamide, 1 mg/mL Xylene Cyanol, 1 mg/mL bromophenol blue, and 20 mM EDTA) followed by 5 min of incubation at 95 °C. The reaction mixture was immediately kept on ice for 5 min. These were then loaded onto 20% (w/v) denaturing PAGE gel containing 8 M urea. The electrophoresis was performed in 1x TBE buffer and the products resolved on the gel were visualized using a Typhoon scanner (GE Healthcare). The quantification of the observed bands was done using ImageQuant TL, 1D gel analysis software^78^.

**Table 2:** Oligonucleotides used in this study are mentioned below in the table.

Name (size)	Oligonucleotides
T1 (50mer)	5′TCCTACCGTGCCTACCTGAACAGCTGGTCACATAAATGCCTACGAGTACG3′
T2 (50mer)	5′TCCTACCGTGCCTACCTGAACAGCTGGTCACATATATGCCTACGAGTACG3′
T3 (50mer)	5′TCCTACCGTGCCTACCTGAACAGCTGGTCACATAGATGCCTACGAGTACG3′
T4 (50mer)	5′TCCTACCGTGCCTACCTGAACAGCTGGTCACATACATGCCTACGAGTACG3′
40A (40mer)	5′TAATCATAAGTATCAGGACTCTCTCTCTCTCAACGGGGGG3′
40C (40mer)	5′TAATCATAAGTATCCGGACTCTCTCTCTCTCAACGGGGGG3′
26 T (26mer)	5′CGGACTCTCTCTCTCTCAACGGGGGG3′
P1 (15mer)	5′**X**CGTACTCGTAGGCAT3′
P2 (34mer)	5′_**Y**_TATGTGACCAGCTGTTCAGGTAGGCACGGTAGGA3′
26P (26mer)	5′**X**CCCCCCGTTGAGAGAGAGAGAGTCCG3′
13P (13mer)	5′_**Y**_ATACTTATGATTA3′
T5 (51mer)	5′TCCTACCGTGCCTACCTGAACAGCTGGTCACATATTATGCCTACGAGTACG3′
T6 (51mer)	5′TCCTACCGTGCCTACCTGAACAGCTGGTCACATATCATGCCTACGAGTACG3′
T7 (51mer)	5′TCCTACCGTGCCTACCTGAACAGCTGGTCACATATAATGCCTACGAGTACG3′
P3 (16mer)	5′**X**CGTACTCGTAGGC_s_A_s_TG3′
P4 (16mer)	5′**X**CGTACTCGTAGGC_s_A_s_TZ3′
P5 (26mer)	5′**X**CCCCCCGTTGAGAGAGAGAGAGTC_s_CU3′
LC (34mer)	5′**X**CGTACTCGTAGGCAGTCGCGAGACCAGCTGTTCG3′

The primers P3, P4, and P5 have phosphorothioate linkages between the mentioned nucleotides at 3′ end which are represented by subscript “s”. X, Y, and Z represent 6FAM, 5′phosphate group, and 8oxodG, respectively.

**Table 3:** The DNA substrates prepared for different biochemical assays performed in this study by annealing the oligonucleotides listed in Table 2.

Oligonucleotides combined (Name)	DNA Substrates
T1/P1/P2(dA)	5′TCCTACCGTGCCTACCTGAACAGCTGGTCACATA**A**ATGCCTACGAGTACG3′ 3′AGGATGGCACGGATGGACTTGTCGACCAGTGTAT_**Y**_-TACGGATGCTCATGC**X**5′
T2/P1/P2 (dT)	5′TCCTACCGTGCCTACCTGAACAGCTGGTCACATA**T**ATGCCTACGAGTACG3′ 3′AGGATGGCACGGATGGACTTGTCGACCAGTGTAT_**Y**_-TACGGATGCTCATGC**X**5′
T3/P1/P2 (dG)	5′TCCTACCGTGCCTACCTGAACAGCTGGTCACATA**G**ATGCCTACGAGTACG3′ 3′AGGATGGCACGGATGGACTTGTCGACCAGTGTAT_**Y**_-TACGGATGCTCATGC**X**5′
T4/P1/P2 (dC)	5′TCCTACCGTGCCTACCTGAACAGCTGGTCACATA**C**ATGCCTACGAGTACG3′ 3′AGGATGGCACGGATGGACTTGTCGACCAGTGTAT_**Y**_-TACGGATGCTCATGC**X**5′
40C/26P	5′TAATCATAAGTATCCGGACTCTCTCTCTCTCAACGGGGGG3′ 3′ GCCTGAGAGAGAGAGAGTTGCCCCCC**X**5′
40C/26P/13P	5′TAATCATAAGTAT**C**CGGACTCTCTCTCTCTCAACGGGGGG3′ 3′ATTAGTATTCATA_**Y**_-GCCTGAGAGAGAGAGAGTTGCCCCCC**X**5′
26 T/26P	5′CGGACTCTCTCTCTCTCAACGGGGGG3′ 3′GCCTGAGAGAGAGAGAGTTGCCCCC**X**5′
T5/P3/P2 (T:G)	5′TCCTACCGTGCCTACCTGAACAGCTGGTCACATATTATGCCTACGAGTACG3′ 3′AGGATGGCACGGATGGACTTGTCGACCAGTGTAT_**Y**_-GT_s_A_s_CGGATGCTCATGC**X**5′
T6/P3/P2 (C:G)	5′TCCTACCGTGCCTACCTGAACAGCTGGTCACATATCATGCCTACGAGTACG3′ 3′AGGATGGCACGGATGGACTTGTCGACCAGTGTAT_**Y**_-GT_s_A_s_CGGATGCTCATGC**X**5′
T6/P4/P2 (C:8oxodG)	5′TCCTACCGTGCCTACCTGAACAGCTGGTCACATAT**C**ATGCCTACGAGTACG3′ 3′AGGATGGCACGGATGGACTTGTCGACCAGTGTAT_**Y**_-ZT_s_A_s_CGGATGCTCATGC**X**5′
T7/P4/P2 (A:8oxodG)	5′TCCTACCGTGCCTACCTGAACAGCTGGTCACATAT**A**ATGCCTACGAGTACG3′ 3′AGGATGGCACGGATGGACTTGTCGACCAGTGTAT_**Y**_-ZT_s_A_s_CGGATGCTCATGC**X**5′
40A/P5/13P (A:U)	5′TAATCATAAGTATCAGGACTCTCTCTCTCTCAACGGGGGG3′ 3′ATTAGTATTCATA_**Y**_-U_s_CCTGAGAGAGAGAGAGTTGCCCCCC**X**5′
40C/P5/13P (C:U)	5′TAATCATAAGTATCCGGACTCTCTCTCTCTCAACGGGGGG3′ 3′ATTAGTATTCATA_**Y**_-U_s_CCTGAGAGAGAGAGAGTTGCCCCCC**X**5′
T3/P1 (R)	5′TCCTACCGTGCCTACCTGAACAGCTGGTCACATA**G**ATGCCTACGAGTACG3′ 3′TACGGATGCTCATGC**X**5′

The names of the DNA substrates prepared are mentioned in the brackets in column 1. The gap in the 1 nucleotide gapped DNA substrates is shown by a hyphen.

### Steady-state kinetic analysis of DNA polymerization on 1 nucleotide gapped molecules

For steady-state kinetics analysis, the ratio of protein: DNA was maintained between 1: 10 and 1: 5. The reaction time where approximately 20% product formed was determined for each DNA substrate and incoming nucleotide combination. The reaction mixture containing 25 mM HEPES pH 7.5, 50 nM-200 nM of DNA substrate, 5 mM MgCl_2_, 0.05 mg/ml BSA, 1 mM of DTT and 5 nM-40 nM of H435A mutant was incubated at 37 °C for 5 min. This pre-incubated reaction mixture was directly added to varying amounts of incoming nucleotide to initiate the polymerization reaction. The reactions were quenched at a predetermined time by the addition of a stop solution followed by 5 min of incubation at 95 °C. The reaction mixture was immediately kept on ice for 5 min and was resolved on 20% (w/v) denaturing PAGE containing 8 M urea. The electrophoresis was performed in 1x TBE buffer and the products resolved on the gel were visualized using a Typhoon scanner (GE Healthcare). The quantification of the observed bands was done using ImageQuant TL, 1D gel analysis software. The steady-state kinetic parameters- K_M_, k_cat_ and catalytic efficiency for the incorporation of nucleotides were determined by fitting the data to the Lineweaver–Burk plot as done in the previous studies^79^. All the experiments were done in triplicates and the standard deviation values were measured.

### Exonuclease assays

Exonuclease assay was performed on 1 nucleotide gapped DNA substrate. The templates and primers used in this study are listed in Table 2. The assays for checking the effect of metal ions were performed with EDTA treated protein. The constituted substrates are listed in Table 3. The reaction mixture (20 μl) comprised of 25 mM HEPES pH 7.5, 50 nM of DNA substrate, 0.05 mg/ml BSA, and 5 mM MgCl_2_ (or MnCl_2_) and 50 nM of wild type saPolX was incubated at 37 °C for 120 min. After incubation for 2 h, the reactions were terminated by the addition of 10 *μ*L of stop solution (80% formamide, 1 mg/mL Xylene Cyanol, 1 mg/mL bromophenol blue, and 20 mM EDTA) followed by 5 min of incubation at 95 °C. The reaction mixture was immediately kept on ice for 5 min. After stopping the reaction, an equal volume of single-stranded oligonucleotide (34mer) loading control (LC) was added into each reaction tube at the final concentration of 50 nM. The loading control had 6FAM label at its 5′-end. These reaction mixtures were analyzed by 20% denaturing PAGE containing 8 M urea in a similar manner as for polymerase assays. For quantifying the degradation of the DNA substrate, the intensity of the substrate band was compared to that of LC for all.

For comparing the time-dependent exonuclease activity of wt-saPolX and H435A mutant, different DNA oligonucleotide templates were annealed to a short primer (5′-end 6FAM labelled and containing phosphorothioate linkages between the second and third nucleotides and third and fourth nucleotide at the 3′ end) (P3 or P4 or P5) at their 3′ end and with primer (P2 or 13P) at their 5′ end to create the gapped substrates. The different templates and primers used in this study are listed in Table 2. The constituted gapped substrates named C:G, T:G, C:8oxodG, A:8oxodG, A:U and C:U are listed in Table 3. For time-course experiments, reaction mixtures comprised of 25 mM HEPES pH 7.5, 50 nM of DNA substrate, 0.05 mg/ml BSA, 5 mM MnCl_2_ and 12.5 nM-50 nM of saPolX (wild type or H435A mutant) was incubated at 37 °C. The reactions were stopped at different time points between 0 and 120 min using the stop solution and by heating and instant cooling. An equal volume of loading control (LC) was added into each reaction tube at the final concentration of 50 nM. For analyzing and quantifying the reaction mixture we used the same method as for the exonuclease assays. All the graphs were prepared using GraphPad Prism 8.0.2. All the experiments were conducted in triplicates and the standard deviation values were measured.

## Supplementary Information

Supplementary Information
