# Supplementary material for: The PHP domain of PolX from Staphylococcus aureus aids high fidelity DNA synthesis through the removal of misincorporated deoxyribo-, ribo- and oxidized nucleotides
Source: Sci Rep. 2021 Feb 18;11:4178. doi: 10.1038/s41598-021-83498-1 (PMC7893174; doi:10.1038/s41598-021-83498-1)
Supplement: Supplementary file 1 — Supplementary Information [file 41598_2021_83498_MOESM1_ESM.pdf]

The PHP domain of PolX from *Staphylococcus aureus* aids high fidelity DNA synthesis through the removal of misincorporated deoxyribo-, ribo- and oxidized nucleotides.

Author: Shilpi Nagpal[1, 2]

Corresponding author: Deepak T. Nair[2]

[1]National Centre for Biological Sciences, Tata Institute of Fundamental Research, GKVK Campus, Bangalore 560065

[2]Regional Centre for Biotechnology, 3rd Milestone, Faridabad-Gurgaon Expressway. Faridabad, 121001

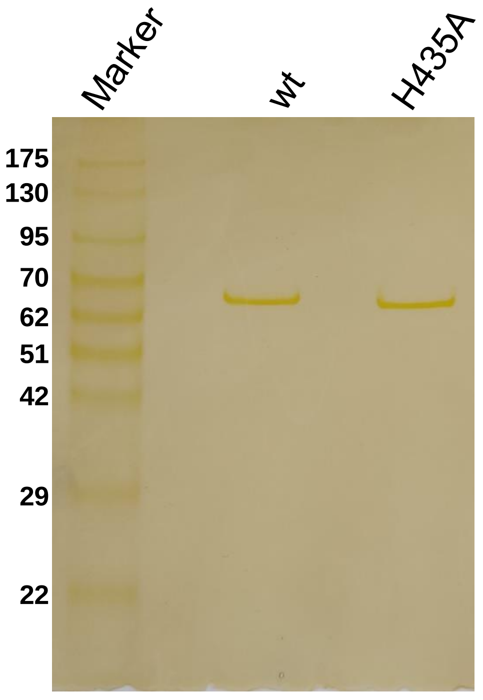

Supplementary Figure S1. Purified saPolX-wt and exonuclease mutant H435A. The purified proteins were run on 12% SDS-PAGE and protein purity was analyzed by Silver staining of the gel. The theoretical molecular weight of saPolX-wt and H435A are 66977.7 Da and 66911.6 Da respectively. 400 ng of each protein sample was loaded on the gel. Both the proteins, wt and H435A mutant show high purity with visibly no contaminants present in the gel. Standard molecular weight markers are labelled in kDa.

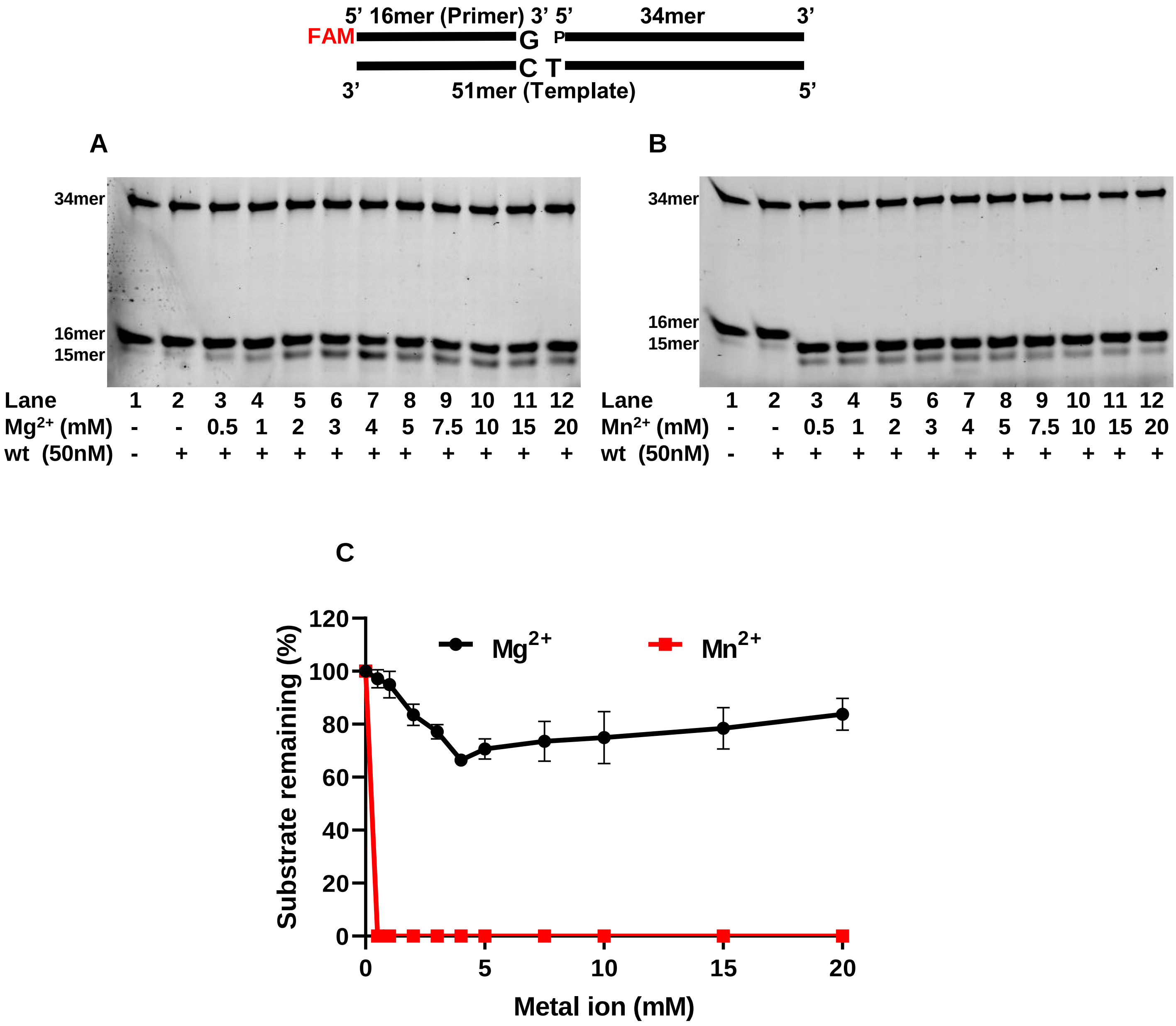

Supplementary Figure S2. Metal ion concentration dependent exonuclease activity of wt-saPolX. The metal ion concentration dependent exonuclease activity was tested in the presence of Mg^{2+} **(A)** and Mn^{2+} **(B)** metal ions for wt-saPolX (50 nM) which was purified with 0.2 M EDTA. The gapped DNA substrate (C:G) used is displayed at the top of the gel. LC represents the loading control (6FAM labelled 34mer DNA oligonucleotide) added in each lane for quantification. The results prove that wt-saPolX shows higher exonuclease activity in the presence of Mn^{2+} than in the presence of Mg^{2+} **(C)**. The maximum exonuclease activity in the presence of Mg^{2+} is 20% which decreases as the concentration of Mg^{2+} increases. In comparison, the exonuclease activity observed in the presence of Mn^{2+} metal ions is 100%. The experiments show that Mn^{2+} metal ion is the metal of choice to exhibit exonuclease activity.
